# Genetic and epigenetic markers in the *METTL21C* gene associated with umbilical hernia in pigs

**DOI:** 10.1186/s12864-025-12315-0

**Published:** 2025-11-18

**Authors:** Jakub Wozniak, Alicja Szabelska-Beresewicz, Joanna Zyprych-Walczak, Rafal Niemyjski, Klaudia Dudek, Monika Stachowiak, Joanna Nowacka-Woszuk

**Affiliations:** 1 Department of Genetics and Animal Breeding, Poznan University of Life Sciences, Wolynska 33, Poznan, 60-637 Poland; 2 Department of Mathematical and Statistical Methods, Poznan University of Life Sciences, Wojska Polskiego 28, 60-637 Poznan, Poland; 3 AgriPlus LLC, Marcelinska 92, 60-324 Poznan, Poland

**Keywords:** RNA-seq, Hernia, Gene expression, DNA methylation, SNP, CNV

## Abstract

**Background:**

Hernias, particularly umbilical hernias (UH), are prevalent anatomical anomalies in swine, leading to significant welfare issues and economic losses. Besides environmental factors also genetic components contribute to the development of UHs, though the exact mechanisms remain unclear. This study employed a multiple approaches integrating RNA-seq, DNA methylation analysis, Sanger sequencing, droplet digital PCR and western blot analysis to investigate the genetic and epigenetic underpinnings of UH in pigs. Muscle tissue from affected and control pigs was examined to identify differentially expressed genes (DEGs) and associated pathways.

**Results:**

We found 59 significant DEGs, including *SIM1*, *PITX1*, *HOXA7*, *METTL21C*, *PVALB*, *ALX1*, *EYA2*, and *TBX1*. Interestingly, RNA-seq analysis of *METTL21C* revealed its significant upregulation in UH-affected pigs. This was corroborated by epigenetic analysis, which identified hypomethylation at four CpG sites in the *METTL21C* within potential regulatory region, aligning with increased mRNA levels. Furthermore, Sanger sequencing uncovered an SNP (rs330073569) in the *METTL21C* regulatory region, which was significantly associated with UH condition. This SNP can potentially alter transcription factor binding leading to enhanced *METTL21C* transcription, and putatively contributing to the gene’s increased expression in UH pigs.

**Conclusions:**

This study highlights potential genetic and epigenetic factors in UH etiology. The most significant result suggests that *METTL21C* plays an important role in the development of UH. This finding makes this gene a promising candidate for further research aimed at better characterizing umbilical hernia in pigs and at potentially eliminating undesirable variants from the gene pool.

**Supplementary Information:**

The online version contains supplementary material available at 10.1186/s12864-025-12315-0.

## Background

Hernia is a common anatomical anomaly encountered within the swine industry [1]. These abnormalities are a type of body wall defect (BWD) and encompass a spectrum of congenital irregularities. BWDs can range from straightforward cases to more intricate conditions [2]. Umbilical hernias (UH) are among the more frequently BWDs observed in pigs [3], and involves the protrusion of abdominal contents, such as intestines, into the hernial sac located in the umbilical region: a ball-like structure under the skin [1]. The general consensus suggests that this condition is caused by the weakness of supportive muscles surrounding the umbilical stump or navel region, leading to improper closure of the umbilical opening. This allows the intestines to protrude through the abdominal wall, which poses a risk of entrapment [4].

This disorder can significantly affect the welfare of pigs and result in significant economic losses. Affected pigs often experience increase mortality and morbidity from infections. Hernias additionally contribute to reduced feed efficiency, decrease pork quality, and pose a risk of carcass contamination with *Salmonella,* which requires additional medical procedures such as herniorrhaphy [2, 5]. Research aimed at reducing the risk of hernia is thus capable of yielding significant positive results in terms of welfare and the economy [5].

Studies point to varying frequencies of umbilical hernias, ranging from 0.4% to 1.5% [6, 7]. Such differences may be due to factors such as breed, sex, line, farm, and production system [7, 8]. Environmental factors also are considered to have a significant impact on hernia development, including infections, nutrition, injuries, management practices, and excessive pressure in the abdomen [4, 9]. On the other hand, a recently published study estimated the heritability of the condition at approximately 0.5 in a mixed population of Duroc, Landrace, and Yorkshire pigs (0.51 ± 0.04) [10], suggesting that genetic components also play a role in its occurrence [5]. This estimate is not universal across all populations and should be interpreted with caution. Other reports suggest considerably lower heritability estimates for UH. Grindflek et al. [4] found that in Norwegian Landrace pigs, the heritability was as low as 0.065. This variability may arise due to differences in breed-specific genetic architecture, environmental pressures and methodological factors such as phenotyping accuracy or the statistical models employed. Thaller et al. [11] demonstrated through simulation studies that the mode of inheritance for rare binary traits such as UH may range from monogenic and digenic to polygenic or mixed models. They showed that the choice of inheritance model and the structure of the data significantly affect the accuracy of parameter estimation and heritability inference. Thus, the mode of inheritance and the underlying mechanisms responsible for the development of umbilical hernias remain elusive, despite attempts to elucidate them. Aside from the influence of environmental factors, it is known that numerous genes interact in the pathogenesis of UH [2]. On the other hand, studies have shown that in some cases, UH may follow a recessive pattern of inheritance [12]. Understanding the genetic mechanisms associated with the emergence of these anomalies is crucial, especially since umbilical hernia is typically not observed at birth; instead, this defect becomes apparent during the pigs’ growth phase. This makes it difficult to eliminate it from the population [13]. Taken together, these findings underscore that heritability estimates of UH vary widely depending on genetic background, environmental conditions and analytical approach. Ding et al. [1] conducted the first genomic study using 194 microsatellite markers, revealing a significant linkage between markers and UH on twelve different chromosomes. Subsequent research, including a genome-wide association study by Grindflek et al. [4], identified new potential genes and single nucleotide polymorphisms (SNPs). Moreover, Long et al. [14] suggested that copy number variation (CNV) on SSC14 might be linked to the development of UH in pigs. One of the recent strategies in research on umbilical hernia in pigs involves identifying gene expression differences that may underlie specific phenotypic changes. A major advancement in this area has been the use of RNA sequencing (RNA-seq), a next-generation sequencing technique. Some of the earliest studies in pigs using this approach focused on inguinal hernia. Researchers analyzed tissues collected from the inguinal ring and identified 703 differentially expressed genes. Pathway analysis revealed that these genes were mainly involved in calcium signaling, apoptosis and smooth muscle differentiation. Ultimately, *BOK*, *DES*, *FGF1*, *MYBPC1*, *MAP1CL3C*, *SLC25A4*, *SLC8A3* and *TPM2* genes were highlighted as strong candidates in hernia pathogenesis [15]. In another study focused on umbilical hernias in pigs, Souza et al. [9] examined tissue samples from the umbilical ring of 10 Landrace pigs: five affected by hernia and five healthy controls. Through RNA-seq, they detected the expression of over 13,000 genes, among which 230 showed significant differential expression. Specifically, 145 genes were downregulated and 85 were upregulated in hernia-affected animals. The altered genes were mainly associated with key biological pathways, including immune system activity, extracellular matrix remodeling, anatomical development, collagen turnover and cell adhesion. Several genes and gene families, such as *ACAN*, *CCBE1*, *COLs*, *EPYC*, *LGALS3*, *MMPs* and *VIT* were highlighted as strong candidates potentially contributing to hernia formation. However, the study was limited by a small sample size and the heterogeneous composition of the analyzed tissues, which included both muscle and connective tissue cells.

In our previous studies, we investigated variants in candidate genes (*OSM* and *ITGAM*) in two large cohorts of UH pigs and found specific haplotypes that reduced the risk of UH [16]. We also found that significant reductions in the transcript level of *MMP13* in connective tissue could potentially predispose to hernia development [17].

While a number of studies on UH have already been carried out, and some genomic regions associated with umbilical hernia have already been identified, the complex nature of this condition seems to be influenced by a multitude of genes and causal variations. Previous research suggests that the incidence of UH is largely affected by breed and population. Moreover, it is known that gene profile expression can differ between tissues. Therefore, to identify the genes and pathways associated with the development of umbilical hernia, we have here conducted an RNA-seq analysis on both normal and affected crossbred pigs using muscle tissue dissected from the umbilical ring. While previous studies have frequently examined the umbilical cord or connective tissues, we specifically selected muscle tissue surrounding the umbilical ring for our analyses. This tissue plays a crucial role in the development of the defect—abdominal muscle weakness can lead to hernia formation [18, 19]. However, in this study, our focus on muscle tissue was intentional to provide clearer insights into how muscle tissue contributes to umbilical hernia. Furthermore, to identify potential causes of the observed changes in expression profiles, we carried out 5’-flanking regions Sanger sequencing and DNA methylation analysis, and we also determined muscular protein level using western blot and droplet digital PCR (ddPCR) for potential CNVs covering the gene of interest.

## Methods

### Ethics statement

The samples in this project were collected during routine veterinary procedures. This case is covered by Polish law and did not require the approval of the local Bioethical Commission for Animal Care and Use in Poznan, Poland (13/01/2020). All experiments were carried out in compliance with the ARRIVE guidelines and all methods were performed in accordance with the relevant guidelines and regulations (including Directive 2010/63/EU).

### Animals, phenotypes, and sample collection

A total of sixty-eight piglets were used in a case–control study. The animals were derived from a cross between a Landrace or Duroc paternal lines and DunBred (Landrace × Yorkshire) maternal hybrid line. All animals came from the same commercial breeding farm located in Wielkopolska Province, Poland. The animals were of both sexes and were 3–4 weeks of age; they were classified by veterinarian into two groups: 34 animals with umbilical hernia constituted the case group, while the 34 healthy pigs (individuals from the same litter as the hernia cases but without any signs of hernia) were included into control group. This resulted in 34 full or half sibling pairs, ensuring each affected animal had a healthy sibling (Additional File 1, Table S1). Most of these pairs were born to different sows, except for five litters (from five sows), where samples were collected from two hernia cases and two healthy controls. Both the hernia-affected and control groups consisted of 34 animals each, comprising 9 castrated boars and 25 sows, respectively. Detailed sample distribution is provided in Additional File 1, Table S1. Euthanasia (without any chemical used) was performed by the percussive stunning (headshot) which rely on a single blow to the head delivered via a captive bolt pistol caused immediate unconsciousness. Next the collection of muscle tissue samples dissected from around the umbilicus of both normal and hernia-affected animals was performed. Tissue samples were promptly frozen in dry ice and stored at −80 °C for subsequent analysis.

### RNA extraction for sequencing analysis

Total RNA extraction from muscle tissue was carried out using a RNeasy Fibrous Tissue Mini Kit (Qiagen) for fifteen affected animals and fifteen controls, representing different litters. Since the umbilical hernia can be the result of weakness of muscle tissue around the navel, this tissue type was selected for the analysis. The fragment of muscle tissue near the site of hernia (around the navel) was dissected carefully to avoid contamination with connective or fat tissue. About 30 mg of tissue from each sample was homogenized in buffer RTL buffer using TissueLyser LT (Qiagen) following the manufacturer’s instructions. For each sample, quality and quantity were rigorously checked. Purity of RNA isolates was measured with a NanoDrop 2000 spectrophotometer (Thermo Fisher Scientific), while RNA concentration was determined using the Qubit RNA BR Assay Kit (Thermo Fisher Scientific) on a Qubit 2.0 fluorometer (Invitrogen). RNA integrity numbers (RIN) were assessed using the RNA 6000 Nano Kit on an Agilent 2100 Bioanalyzer (Agilent Technologies). Samples with RIN values exceeding a threshold of 8 and with 260/280 nm ratio above 2.0 were selected for downstream analysis. For library construction, 100 ng of total RNA per sample was processed using the TruSeq Stranded mRNA Kit (Illumina). Sequencing was performed by an external service on an Illumina NovaSeq 6000 platform, generating paired-end reads of 2 × 100 bp. Raw sequenced fragments ranged from 27.863 to 83.380 million per sample, with a mean of approximately 40 million reads. The sequencing quality was confirmed by ensuring a Q30 value greater than 90%. The mapping rate achieved an average of approx. 95% properly paired reads. The resulting sequencing data were deposited in the NCBI Sequence Read Archive (SRA) database (accession number: PRJNA1147879).

### Bioinformatic analysis of RNA-seq results

The sequencing data quality was meticulously evaluated using FastQC [20]. Adapter sequences were removed using BBDuk2 from the BBmap suite (v. 36.32, https://sourceforge.net/projects/bbmap/), and low quality reads (Phred quality score < 5) were clipped from both ends. The cleaned reads were then aligned to the pig reference genome (Sus_scrofa.Sscrofa11.1.dna.toplevel.fa.gz, Ensembl database) using the STAR v. 2.5.3 [21] tool with default parameters and gtf file (Sus_scrofa.Sscrofa11.1.104.gtf, Ensembl database). Read counts were obtained using the Rsubread package v. 1.32.4 [22]. For the subsequent analysis, genes with low expression—defined as counts per million (CPM) below 0.5 in at least two samples—were filtered out to ensure robustness. Data normalization was conducted using the Trimmed Mean of M-values (TMM) method, as implemented in the edgeR package v. 4.0.16 [23]. Differential expression analysis was performed using a generalized linear model provided by the limma package v. 3.58.1 [24]. The fundamental grouping variable in the model was the disease status. Because of the correlation between samples resulting from the same mother, we included this relationship in the model. The sex of the animal was also included. This was accomplished using the duplicateCorrelation function of the limma package. The set of differentially expressed genes (DEGs) was determined using two criteria: an adjusted *p*-value (false discovery rate: FDR) < 0.05, and an absolute log₂fold change $\left|{\text{log}}_{2}FC\right|>1$. Only genes that fulfilled both conditions were considered differentially expressed. For the gene ontology (GO) enrichment analysis were used differentially expressed genes that fulfil conditions FDR < 0.05 together with $\left|{\text{log}}_{2}FC\right|>1$. GO analysis was conducted using an enrichment test based on the hypergeometric distribution implemented in systemPipeR Bioconductor package [25]. The statistical significance of GO term enrichment was determined using the Bonferroni adjusted *p-*value < 0.01.

All visualizations were prepared using the ggplot2 [26] and gplots [27] packages. All data preprocessing was performed using the tidyverse ecosystem of R packages [26], and statistical analyses were carried out using R software [28].

### Identification of polymorphisms

For the polymorphism searching, RNA-seq reads obtained from 30 muscle tissue samples (15 umbilical hernia cases and 15 controls) were used. The alignment of sequencing reads to a reference genome of Sus scrofa was conducted using the STAR v. 2.5.3 [21], with parameters that were selected to optimize the analysis of genomic variants. Variant calling was subsequently performed with GATK [29]. Duplicates were marked using Samtools. Identified variants were annotated with the VariantAnnotation and snpStats packages, with low-quality and infrequent variants being excluded from further analysis. The functional impact of each SNPs was predicted using the Ensembl Variant Effect Predictor (VEP) version 104. Annotation allowed identification of novel polymorphisms and helped confirm their locations and potential functions within the genome. Differences in SNP frequencies between groups were statistically analysed using Fisher’s exact test, with FDR adjustment to account for multiple testing.

### RNA extraction and cDNA synthesis for validation

The RNA extraction was performed using a RNeasy Fibrous Tissue Mini Kit (Qiagen), as described previously, for RNA-seq protocol for 34 affected animals and 34 controls. The quantity and quality of isolate were evaluated using a NanoDrop ND-2000 spectrophotometer (Thermo Fisher Scientific). The cDNA was synthesized from 1 μg of RNA using the Transcriptor First Strand cDNA Synthesis Kit (Roche) in a 20 μL reaction volume. Finally, the cDNA was diluted with nuclease-free water to a final concentration of approximately 700 ng/µL.

### Validation of RNA-seq results using quantitative polymerase chain reaction (qPCR)

We employed the qPCR method to confirm the RNA-seq results in a group of 34 affected animals and 34 controls. This included samples from the pigs used in the RNA-seq analysis. Eleven genes for validation were selected based on significant adjusted* p-*value, and logFC score from the RNA-seq data. We also considered whether the selected genes were involved in specific biological pathways important for proper muscle tissue function, which were significant in Gene Ontology analysis. Moreover, the function of each was considered according to available databases. The chosen genes were: SIM bHLH transcription factor 1 (*SIM1*), Paired Like Homeodomain 1 (*PITX1*), Homeobox A7 (*HOXA7*), Methyltransferase 21 C (*METTL21C*), Iroquois Homeobox 5 (*IRX5*), Parvalbumin (*PVALB*), ALX Homeobox 1 (*ALX1*), ALX Homeobox 4 (*ALX4*), EYA Transcriptional Coactivator And Phosphatase 2 (*EYA2*), T-Box Transcription Factor 1 (*TBX1*), and Odd-Skipped Related Transcription Factor 2 (*OSR2*). Primers were designed using Primer3Plus software (version 3.2.0; https://www.primer3plus.com; accessed on 2 March 2023; Additional File 1, Table S2). A LightCycler 480 SYBR Green I Master (Roche) was used to performed qPCR in a total reaction mix of 10 μL per well. The reactions for each gene were performed following the manufacturer’s instructions in three repetitions. The thermocycler protocol commenced with an initial denaturation phase at 95 °C for ten minutes, succeeded by 45 cycles consisting of denaturation at 95 °C for ten seconds, annealing at 60 °C for five seconds, and elongation at 72 °C for five seconds. Subsequent to each amplification, a melting curve analysis was conducted to confirm product specificity. The relative expression levels of the target genes were determined utilizing the standard curve method, employing a set of ten-fold dilutions of a DNA sample with known concentration (standards). To normalize the relative changes in gene expression, levels were compared with the reference genes *H3F3A* (H3 histone, family 3 A) and *PPIA* (Peptidyl-prolyl cis–trans isomerase A) [30], employing the normalization method outlined by [31]. Transcript levels were then compared between the tested groups using a nonparametric two-tailed Mann–Whitney *U*-test and a post-hoc Dunn’s test with the Benjamini and Hochberg [32] correction (FDR) in R software using the of stats package [28]. The Spearman's rank correlation coefficient for qPCR and RNA-seq results was calculated in R software using stats package [28].

### Methylation analysis by pyrosequencing

The analysis, focused on identifying changes in DNA methylation level, was performed for eight genes that showed significant differences in qPCR. The CpG sites were chosen on the basis of information from the NCBI Gene database and CpGPlot software (version EMBOSS 6.6.0; http://www.ebi.ac.uk/Tools/seqstats/emboss_cpgplot/; accessed on 18 January 2023). The analysis pointed to several CpG sites in the noncoding 5’-flanking regions of *SIM1*, *PITX1*, *HOXA7*, *METTL21C*, *ALX1*, and *EYA2* in the 5′ untranslated region (5′UTR) of the first exon of *TBX1* and in coding region of the first exon of *PVALB* (Additional File 1, Table S3). The primers for the pyrosequencing analysis (Additional File 1, Table S2) were design using PyroMark Assay Design 2.0 software (Qiagen) to amplify the bisulfite modified target regions. DNA was isolated from the muscle tissue of 34 healthy pigs and 34 animals with hernia, using a Genomic Mini Kit (A&A Biotechnology). A NanoDrop ND-2000 spectrophotometer (Thermo Fisher Scientific) with 260/280 nm wavelength readings was used to assess the quantity and quality of isolates. To prepare the samples for the pyrosequencing reaction, the bisulfite conversion was performed with an EZ DNA Methylation-Gold kit (Zymo Research) on 500 ng of DNA; nonmethylated controls were gained using REPLI g Mini Kits (Qiagen), following the manufacturer’s instructions. Additionally, fully methylated controls were prepared by incubating 500 ng of DNA with CpG methyltransferase (M.SssI, Thermo Fisher Scientific) for three hours at 37 °C. In the next step, the PCR reaction was performed using a PyroMark PCR kit (Qiagen) with the protocol as follows: initial denaturation at 95 °C for fifteen minutes; 44 cycles comprising denaturation at 94 °C for thirty seconds, primer annealing for thirty seconds at 56 °C, and elongation at 72 °C for thirty seconds. This was followed by a final extension step at 72 °C for ten minutes. Additionally, negative control samples (lacking a DNA template) were included in each reaction. The efficiency of PCR was determined using electrophoresis on 1.5% agarose gel. The pyrosequencing of the amplicons was performed using a PyroMark Q48 Autoprep system (Qiagen) with PyroMark Q48 Advanced CpG Reagents (Qiagen), following the protocol. The percentage of methylation at each CpG site was subsequently calculated using the PyroMark Q48 Autoprep software (Qiagen). The CpG methylation levels (%) were compared between the affected and control groups using a nonparametric two-tailed Mann–Whitney *U*-test with the FDR correction in R software using the of stats package [28]. The Spearman's rank correlation coefficient for DNA methylation and qPCR results was calculated in R software using stats package [28]. The analysis was conducted exclusively for CpG sites that showed a significant difference in methylation level.

### DNA sanger sequencing analysis of 5’-flanking regions

The 5’-flanking regions were sequenced for the following eight genes, which showed significant differences in qPCR: *SIM1*, *PITX1*, *HOXA7*, *METTL21C*, *ALX1*, *EYA2, TBX1,* and *PVALB*. DNA was isolated from the muscle tissue of 34 UH pigs and 34 healthy animals using the Genomic Mini Kit (A&A Biotechnology). The primers for PCR were designed using Primer3Plus software (https://primer3plus.com/cgi-bin/dev/primer3plus.cgi; Additional File 1, Table S2) on the basis of the pig genome sequence Sscrofa11.1 for the noncoding 5’-flanking region of each gene. The PCR products, after checking on agarose gel, were purified using alkaline phosphatase and exonuclease (Thermo Fisher Scientific). Sequencing was performed following the manufacturer’s instructions with BigDye Terminator v3.1 Cycle Sequencing Kits (Thermo Fisher Scientific). Finally, the products were filtered through Sephadex G50 (Sigma). Capillary electrophoresis was conducted on a Genetic Analyzer 3500 (Applied Biosystems) and the results were analysed using SeqMan software from the DNASTAR package (DNASTAR). In order to find potential differences with the reference genome (Sscrofa11.1), the resulting sequences were compared to the following reference sequences from GenBank: *SIM1* (GenBank ID: 100,154,026), *PITX1* (GenBank ID: 100,689,266), *HOXA7* (GenBank ID: 100,519,456), *METTL21C* (GenBank ID: 100,525,886), *ALX1* (GenBank ID: 100,525,700), *EYA2* (GenBank ID: 100,624,872)*, TBX1* (GenBank ID: 110,256,621), and *PVALB* (GenBank ID: 100,157,265). Allele counts were compared between the UH and control groups using the odds–ratio test in the MedCalc Software online platform (https://www.medcalc.org/calc/odds_ratio.php). Additionally, transcription factor binding sites in the analysed sequence of *METTL21C* gene were predicted with the use of ALGGEN-PROMO version 8.3 online software (https://alggen.lsi.upc.es/cgibin/promo_v3/promo/promoinit.cgi?dirDB = TF_8.3) [33] and the Neural Network Promoter Prediction (NNPP) tool available at https://www.fruitfly.org/seq_tools/promoter.html was used to confirm that the studied 5’- flanking sequence was a promoter regions. The analysis was conducted with a score cutoff of 0.80, ensuring the identification of high-confidence promoter sequences. To further support the biological relevance of this gene in skeletal muscle, public transcriptomic data such as The Pig RNA Atlas (https://www.rnaatlas.org/) were consulted.

### Sanger sequencing variants identified by RNA-seq

Sanger sequencing genotyped three candidate missense: SNPs rs341151132 in the *GALNT16* gene, rs690269483 in *PAOX*, and rs334837422 in *RFTN1*, as well as one SNP in the 3′ untranslated region (3′UTR), rs318378401 in *SLC9A1*. These SNPs were selected on the basis of the variant analysis of the RNA-seq data and were subsequently analysed for DNA isolated from muscle tissue in the 34 UH and 34 control animals. DNA from muscle was isolated using a Genomic Mini Kit (A&A Biotechnology). Primer design (Additional File 1, Table S2) and sequencing was performed as described the section entitled “DNA sanger sequencing analysis of 5’-flanking regions”. The resulting sequences were compared to the following reference sequences from GenBank: *GALNT16* (GenBank ID: 100,513,992), *PAOX* (GenBank ID: 100,626,281), *RFTN1* (GenBank ID: 100,155,924), and *SLC9A1* (GenBank ID: 397,458). Allele counts were compared between groups using the odds–ratio test on the MedCalc Software online platform (https://www.medcalc.org/calc/odds_ratio.php).

### Droplet digital PCR (ddPCR) analysis

The ddPCR method was used to determine whether copy number polymorphism affected the genes of interest in the 34 affected piglets and 34 controls. The DNA was isolated from muscles using the Genomic Mini Kit (A&A Biotechnology). The assays for gene fragment amplification were designed at the following positions: SSC1: 67,292,924 bp – SSC1: 67,293,017 bp for *SIM1*, SSC2: 137,206,573 bp – SSC2: 137,206,652 bp for *PITX1*, SSC18: 45,417,413 bp – SSC18: 45,417,524 bp for *HOXA7*, SSC11: 71,018,624 bp – SSC11: 71,018,742 bp for *METTL21C*, SSC5: 96,784,832 bp – SSC5: 96,784,939 bp for *ALX1*, SSC17: 49,053,986 bp – SSC17: 49,054,105 bp for *EYA2,* SSC4: 51,290,197 bp – SSC14: 51,290,276 bp for *TBX1,* and SSC5: 10,955,592 bp – SSC5: 10,955,677 bp for *PVALB*. The selected regions, which typically overlapped with exon 1 (often corresponding to the 5′UTR), were chosen to assess potential variation near the 5′ end of the gene that could extend into the promoter region and influence gene expression. The coagulation factor II gene (*F2*) was selected as the reference gene [34] (Additional File 1, Table S2). All procedures were performed following the manufacturer’s instruction. The PCR mixtures were made by combining the gene assay (with the FAM-labelled probe), ddPCR Supermix, an *EcoR*I restriction enzyme, the probe for the reference gene (HEX-labelled), primers, DNA, and water. The QX200 droplet generator (Bio-Rad) divided the PCR mixtures into approximately 20,000 droplets. The samples were then subjected to emulsion PCR on a Gradient T100 Thermal Cycler (Bio-Rad with the following protocol: initial DNA denaturation at 95 °C for 10 min; 40 cycles at 94 °C for 30 s and at 58 °C for 60 s (ramp rate 2 °C/s); the final step was at 98 °C for 10 min. After amplification, the number of DNA copies was detected using a QX200 droplet reader (Bio-Rad). The QuantaSoft software (version 1.7.4.0917, Bio-Rad) was used to analyze the outcomes and calculate the number of copies using the Poisson distribution with two copies of the reference gene (*F2*) as a standard.

### Western blot analysis

Western blot analysis was performed for twelve animals with umbilical hernia and twelve healthy controls. The proteins were isolated from muscle tissue. The fragment of tissue from each sample was homogenized in lysis buffer RIPA (Sigma-Aldrich). To ensure they met the required quality standards for subsequent analysis and quantitative assessment, we used a Protein Assay Kit (Thermo Fisher Scientific) and a Qubit fluorometer (Invitrogen). Only four proteins were selected for analysis because of antibody-specific problems for the pig for other proteins of interest. SIM1 (Abcam; goat antibody ab126863, dilution 1:250), PITX1 (Abcam; rabbit antibody ab70273, dilution 1:500), EYA2 (Thermo Fisher; rabbit antibody PA5-68,561, dilution 1:500) and TBX1 (Abcam; rabbit antibody ab109313, dilution 1:500) were used as primary antibodies. GAPDH (Abcam; mouse antibody ab8245, dilution 1:5000) was used as a reference protein. The samples were prepared by mixing 25 μg of total proteins for SIM1, PITX1, EYA2 and 30 μg for TBX1 with Bolt LDS Sample Buffer (Thermo Fisher) and Bolt Sample Reducing Agent (Thermo Fisher). Follow denaturation at 70 °C for 10 min, the samples were loaded with the same volume on Bolt 12% Bis–Tris Plus gel. Electrophoresis was run at 120 V for 150 min, and the protein was then transferred onto a nitrocellulose membrane (Life Technologies). Five-percent nonfat dried milk (dissolved in TBST buffer) was used to block the membranes at room temperature for 120 min, and was then washed twice with TBST buffer and incubated with the primary antibody overnight at 4 °C. Each sample was loaded onto the gel in duplicate for electrophoresis; one replicate was tested with the specific primary antibodies, while the other replicate was tested with the GAPDH reference antibody. This approach ensured consistency and accuracy in the analysis, allowing for precise normalization of the studied protein against the reference protein. After incubation, the membranes were washed three times with TBST to remove any unbound antibodies. They were then incubated with the appropriate secondary antibody (Abcam; goat anti-rabbit ab97051, dilution 1:5000, Abcam; rabbit anti-mouse ab6728, dilution 1:5000, Abcam; rabbit anti-goat ab6741, dilution 1:5000) conjugated with horseradish peroxidase (HRP) at room temperature for two hours. The final step was detection using an Immobilon Forte Western HRP substrate (Merck) on a ChemiDoc Touch Imaging System (Bio-Rad). The band intensities were analysed using ImageLab software (Bio-Rad) and the results were studied statistically using a nonparametric two-tailed Mann–Whitney *U*-test in R software using the of stats package [28].

## Results

### Global transcript level analysis

**Fig. 1:**
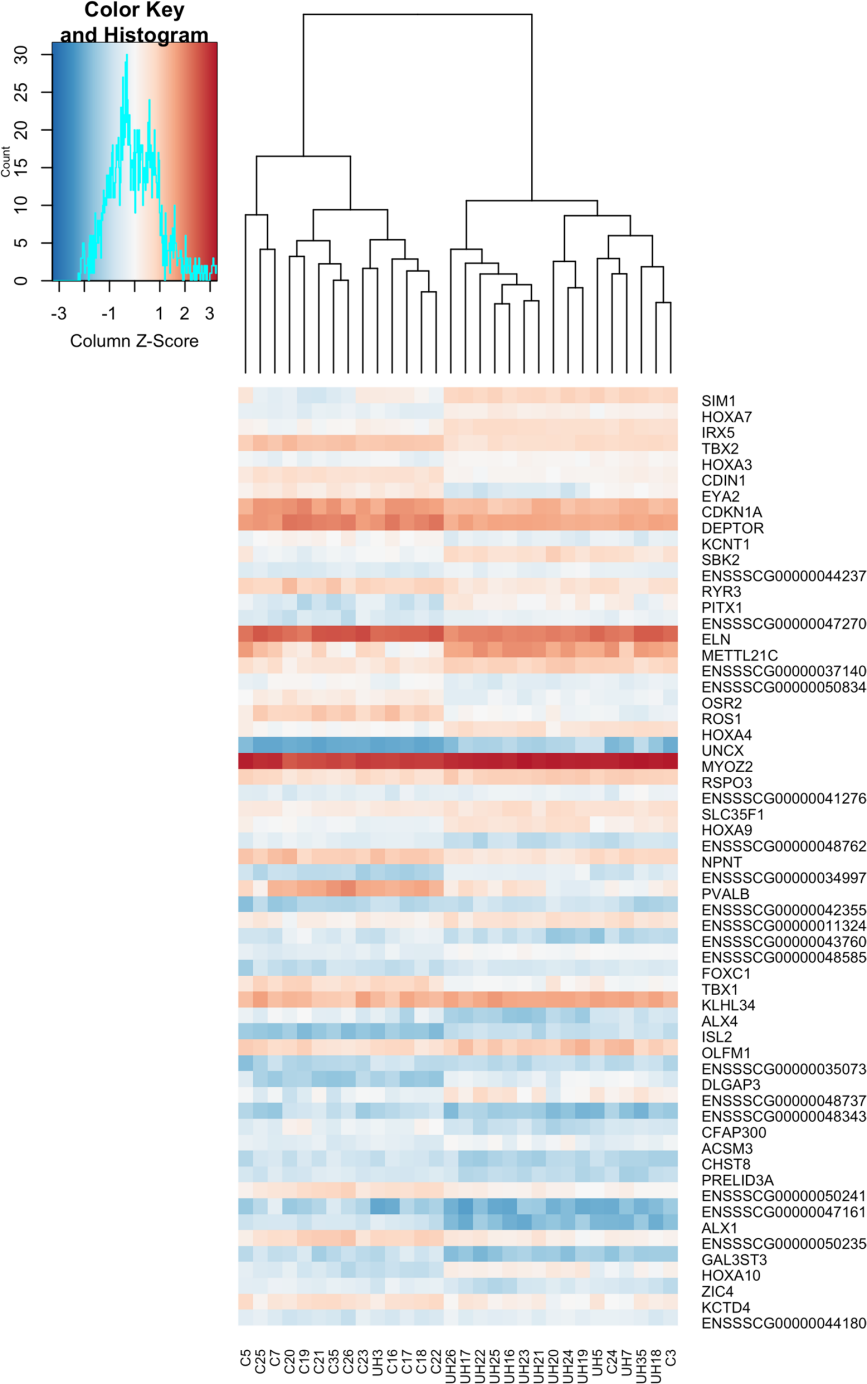
Heatmap representing the all DEGs with best FDR in the control and UH individuals

We conducted an in-depth analysis of the muscular transcriptome from the UH (n = 15) and control animals (n = 15). In total, the expression levels of 14,919 genes were examined (Additional File 2), revealing that 234 genes were statistically significant at the 0.05 significance level. When additional criterium was applied such that FDR < 0.05 and $\left|{\text{log}}_{2}FC\right|>1$, 59 genes showed altered transcript level. Of these, 31 were upregulated (Table 1) and 28 were downregulated (Table 2) for UH individuals compared to controls. Of these in turn, 79.66% of the DE genes were characterized as protein coding genes, 18.64% as lncRNA, and 1.7% as pseudogenes. All DEGs with the best FDRs are shown in Fig. 1.

**Table 1:** The list of 31 upregulated genes with FDR < 0.05 and logFC > 1

Gene	logFC	P_adj_	symbol
ENSSSCG00000004357	3.75	1.8 × 10^−4^	*SIM1*
ENSSSCG00000016701	2.26	1.8 × 10^−4^	*HOXA7*
ENSSSCG00000032145	2.19	2.7 × 10^−4^	*IRX5*
ENSSSCG00000016705	1.31	3.9 × 10^−4^	*HOXA3*
ENSSSCG00000033532	2.62	3.0 × 10^−3^	*SBK2*
ENSSSCG00000044237	1.17	3.0 × 10^−3^	LncRNA
ENSSSCG00000036741	3.11	4.7 × 10^−3^	*PITX1*
ENSSSCG00000047270	1.59	6.0 × 10^−3^	LncRNA
ENSSSCG00000031101	3.63	6.0 × 10^−3^	*METTL21C*
ENSSSCG00000037140	1.43	6.1 × 10^−3^	
ENSSSCG00000016704	1.92	8.8 × 10^−3^	*HOXA4*
ENSSSCG00000007555	3.45	1.0 × 10^−2^	*UNCX*
ENSSSCG00000032838	1.03	1.2 × 10^−2^	*MYOZ2*
ENSSSCG00000004218	1.11	1.2 × 10^−2^	*RSPO3*
ENSSSCG00000041276	1.04	1.2 × 10^−2^	LncRNA
ENSSSCG00000004250	1.01	1.2 × 10^−2^	*SLC35F1*
ENSSSCG00000028997	2.17	1.3 × 10^−2^	*HOXA9*
ENSSSCG00000034997	2.50	1.5 × 10^−2^	
ENSSSCG00000042355	1.47	1.6 × 10^−2^	
ENSSSCG00000011324	1.19	1.7 × 10^−2^	
ENSSSCG00000048585	1.37	1.7 × 10^−2^	LncRNA
ENSSSCG00000039756	1.29	1.7 × 10^−2^	*FOXC1*
ENSSSCG00000033979	1.04	2.0 × 10^−2^	*KLHL34*
ENSSSCG00000033348	2.43	2.1 × 10^−2^	*ISL2*
ENSSSCG00000029715	1.17	2.1 × 10^−2^	*OLFM1*
ENSSSCG00000035073	1.07	2.1 × 10^−2^	
ENSSSCG00000040173	3.77	2.5 × 10^−2^	*DLGAP3*
ENSSSCG00000048737	2.50	2.7 × 10^−2^	LncRNA
ENSSSCG00000007857	1.20	2.9 × 10^−2^	*ACSM3*
ENSSSCG00000021204	3.15	3.2 × 10^−2^	*HOXA10*
ENSSSCG00000044180	1.00	4.7 × 10^−2^	LncRNA

*logFC* Logarithm of Fold Change, *P*_*adj*_ adjusted *p*-value

**Table 2:** The list of 28 downregulated genes with FDR < 0.05 and logFC < −1

Genes	logFC	P_adj_	symbol
ENSSSCG00000027060	−1.18	3.7 × 10^−4^	*TBX2*
ENSSSCG00000007453	−2.28	4.2 × 10^−4^	*EYA2*
ENSSSCG00000026900	−1.27	4.2 × 10^−4^	*CDIN1*
ENSSSCG00000001565	−1.31	1.3 × 10^−3^	*CDKN1A*
ENSSSCG00000049630	−1.20	2.3 × 10^−3^	*DEPTOR*
ENSSSCG00000005762	−1.26	2.8 × 10^−3^	*KCNT1*
ENSSSCG00000033958	−1.18	4.6 × 10^−3^	*RYR3*
ENSSSCG00000025858	−1.04	6.1 × 10^−3^	*ELN*
ENSSSCG00000050834	−1.03	6.1 × 10^−3^	
ENSSSCG00000006073	−1.44	8.1 × 10^−3^	*OSR2*
ENSSSCG00000026978	−2.89	8.1 × 10^−3^	*ROS1*
ENSSSCG00000048762	−1.25	1.3 × 10^−2^	
ENSSSCG00000038801	−1.10	1.4 × 10^−2^	*NPNT*
ENSSSCG00000000138	−3.71	1.6 × 10^−2^	*PVALB*
ENSSSCG00000043760	−1.92	1.7 × 10^−2^	LncRNA
ENSSSCG00000037762	−1.78	1.9 × 10^−2^	*TBX1*
ENSSSCG00000040329	−2.34	2.1 × 10^−2^	*ALX4*
ENSSSCG00000048343	−1.89	2.7 × 10^−2^	Pseudogene
ENSSSCG00000014980	−1.77	2.8 × 10^−2^	*CFAP300*
ENSSSCG00000047161	−2.60	2.9 × 10^−2^	LncRNA
ENSSSCG00000050241	−1.08	2.9 × 10^−2^	LncRNA
ENSSSCG00000036781	−1.03	2.9 × 10^−2^	*PRELID3A*
ENSSSCG00000035222	−1.02	2.9 × 10^−2^	*CHST8*
ENSSSCG00000036744	−2.89	2.9 × 10^−2^	*ALX1*
ENSSSCG00000050235	−1.32	3.0 × 10^−2^	LncRNA
ENSSSCG00000028015	−1.71	3.1 × 10^−2^	*GAL3ST3*
ENSSSCG00000011693	−1.06	4.0 × 10^−2^	*ZIC4*
ENSSSCG00000022868	−1.14	4.3 × 10^−2^	*KCTD4*

*logFC* Logarithm of Fold Change, *P*_*adj*_ adjusted *p*-value

### Analysis of functional enrichment pathway

**Fig. 2:**
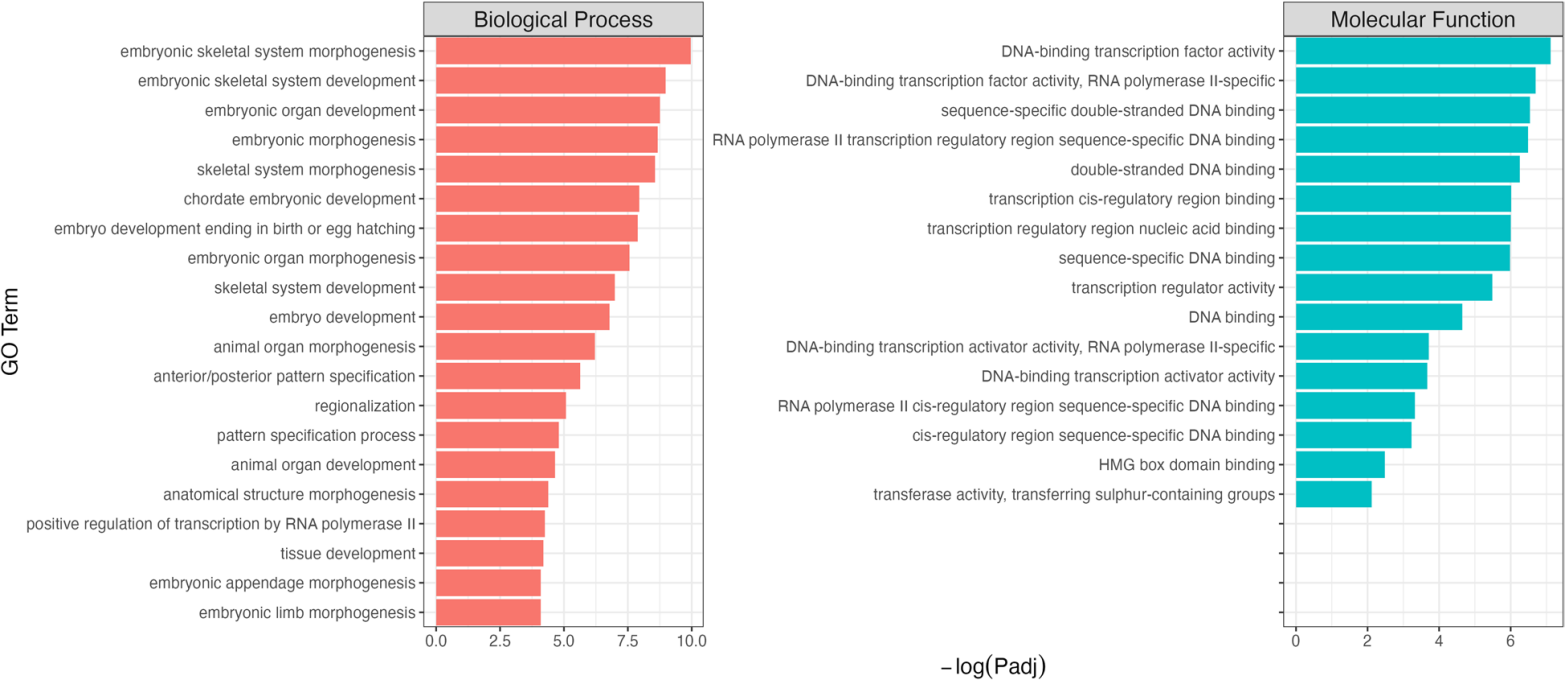
GO enrichment analysis for DEGs. Significantly enriched GO terms (adjusted *p*-values < 0.01) are plotted against -log10 transformed adjusted *p*-values

We evaluated the enrichment of GO terms for DEGs meeting the criteria of FDR < 0.05 and $\left|{\text{log}}_{2}FC\right|>1$. To achieve this, we performed an enrichment analysis using the hypergeometric distribution. The significance threshold for each GO term, as determined by the Bonferroni-corrected *p*-values, was set at 0.01. This rigorous approach ensures that the identified GO terms are highly reliable and relevant to the biological processes under investigation, minimizing the likelihood of false positives and enhancing the robustness of our findings. In UH animals, sixty significant biological processes and sixteen molecular functions were identified (Additional File 3). Notably, these processes encompass critical biological functions and pathways, highlighting the differential gene expression patterns between the conditions. The most significant GO terms ​​(max twenty processes) in each ontology category are shown in Fig. 2. Among the most significant biological processes (BP), embryonic skeletal system morphogenesis stood out, involving genes such as *ALX1*, *HOXA9*, *HOXA7*, *HOXA4*, *HOXA3*, *IRX5, OSR2*, and *ALX4*. The process of embryonic organ development was associated with a broader set of genes, including *ALX1*, *TBX2*, *HOXA9*, *HOXA7*, *HOXA4*, *HOXA3*, *IRX5*, *OSR2*, *FOXC1*, *RSPO3*, and *ALX4*. Similarly, chordate embryonic development included key genes such as *ALX1*, *TBX2*, *HOXA9*, *HOXA7*, *HOXA4*, *HOXA3*, *IRX5*, *OSR2*, *CDKN1A*, *FOXC1*, *RSPO3*, and *ALX4*. Another enriched pathway, embryonic morphogenesis involved the same genes, along with *HOXA10* but without *CDKN1A*.

### Validation of RNA-seq data using quantitative PCR (qPCR)

**Fig. 3:**
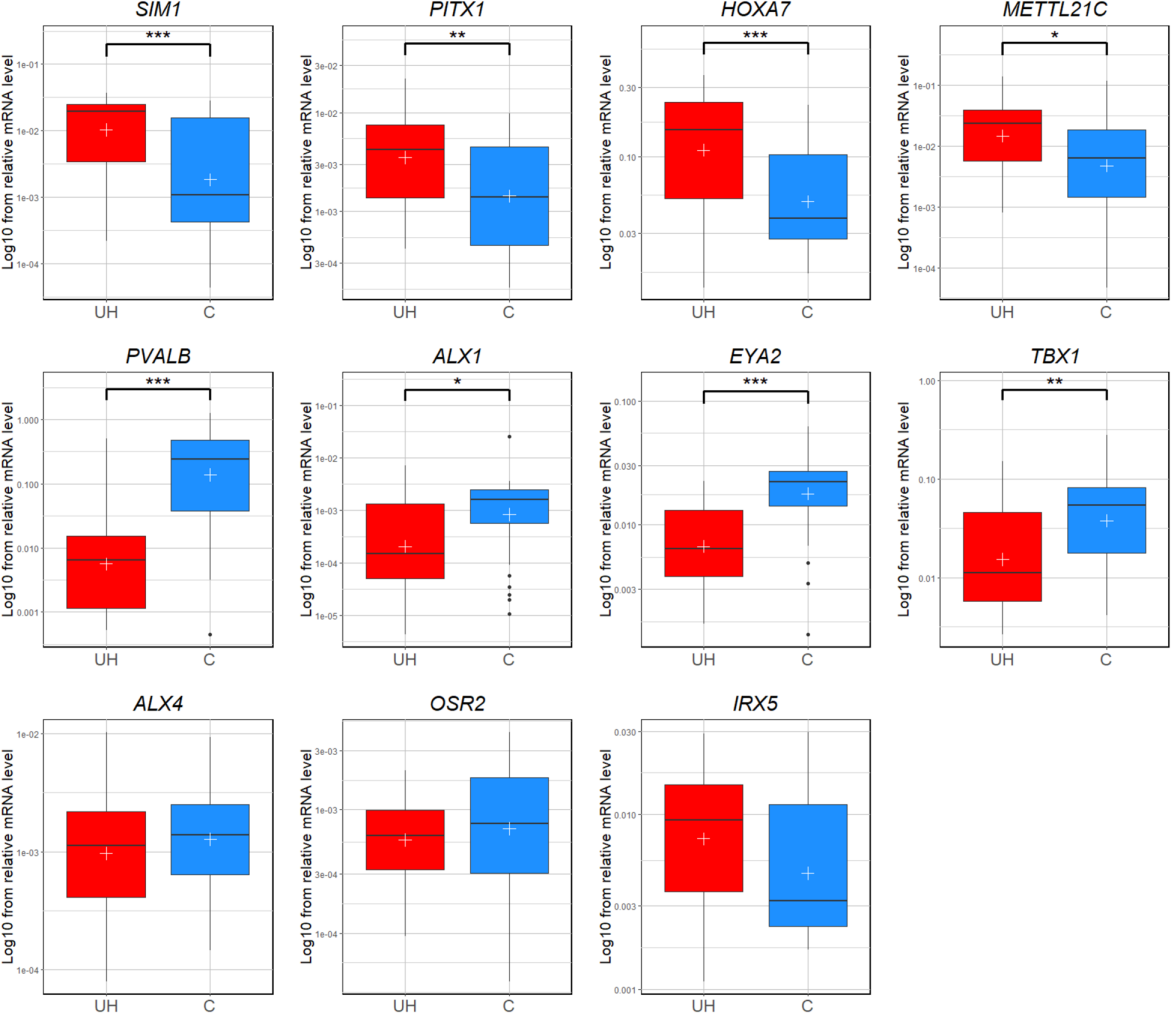
The Log10 of the relative mRNA levels of the *SIM1*, *PITX1*, *HOXA7*, *METTL21C*, *PVALB*, *ALX1*, *EYA2*, *TBX1*, *ALX4*, *OSR2*, and *IRX5* genes obtained from the UH (case) and C (control) pigs. The vertical black lines crossing the boxes show medians and the white crosses represent mean values. The lines below and above the rectangles indicate the maximum and minimum values, while the black dots positioned beneath and above the boxes represent outliers. Asterisks indicate statistical significances between the UH and control groups: one asterisk (*) denotes a *p*-value less than 0.05, two asterisks (**) denote a *p*-value less than 0.01, and three asterisks (***) denote a *p*-value less than 0.001

To validate the results of the RNA-seq analysis, real-time PCR was performed on the same tissue samples collected from both normal and affected pigs. The relative mRNA level of all the validated genes was calculated and the trend of differential expression observed in the qPCR was consistent with the findings from the RNA-seq analysis. The correlation analysis performed on genes that were significant in qPCR demonstrated a high correlation between RNA-seq count data and qPCR results, that were statistically significant for all considered genes (p < 0.001), as indicated by a Spearman’s correlation coefficient ($\rho>0.8$) (Additional File 1, Table S4). The *SIM1*, *PITX1*, *HOXA7*, *METTL21C*, and *IRX5* genes were upregulated in affected pigs in contrast to the controls, while *PVALB*, *ALX1*, *ALX4*, *EYA2*, *TBX1*, and *OSR2* were downregulated (Fig. 3, Table 3). The direction of expression changes was consistent between RNA-seq and qPCR for all analysed genes. The statistical analysis showed significant results (*p* < 0.05) for eight out of eleven studied genes. The most significant differences were observed for downregulated genes, where for *PVALB* and *EYA2* the level of expression in UH group comparing to controls was almost ten and three times decreased, respectively (*p* < 0.0001). It was observed that two other downregulated genes (*TBX1* and *ALX1*) exhibited diminished differences in their mRNA levels between studied groups, but still significant: *p* < 0.01 and *p* < 0.05, respectively. In terms of upregulated genes, the most differential mRNA level was observed for *SIM1* and *HOXA7* genes (*p* < 0.001), while two other genes (*METTL21C* and *PITX1*) had doubled expression in UH compared to control group (*p* < 0.05 and *p* < 0.01, respectively) – Table 3. The transcript level was non-significant for *IRX5, ALX4*, and *OSR2*; these three genes were therefore excluded from further analysis.

**Table 3:** Overview of results of the RNA-seq and qPCR analysis

Genes	RNA-seq		qPCR				
	**logFC**	**P**_**adj**_	**Mean value**		**SD value**		**P**_**adj**_
			**UH**	**C**	**UH**	**C**	
*SIM1*	3.75	1.8 × 10^–4^	0.0173	0.0070	0.0111	0.0096	2.6 × 10^–4^
*METTL21C*	3.63	6.0 × 10^–3^	0.0314	0.0157	0.0345	0.0252	1.2 × 10^–2^
*PITX1*	3.11	4.7 × 10^–3^	0.0054	0.0028	0.0048	0.0029	7.7 × 10^–3^
*HOXA7*	2.26	1.8 × 10^–4^	0.1562	0.0712	0.1088	0.0657	5.0 × 10^–4^
*IRX5*	2.19	2.7 × 10^–4^	0.0106	0.0071	0.0077	0.0073	9.3 × 10^–2^
*OSR2*	−1.44	8.1 × 10^–3^	0.0008	0.0012	0.0006	0.0011	2.1 × 10^–1^
*TBX1*	−1.79	1.9 × 10^–2^	0.0302	0.0633	0.0374	0.0577	7.7 × 10^–3^
*EYA2*	−2.28	4.2 × 10^–4^	0.0086	0.0226	0.0060	0.0143	3.0 × 10^–6^
*ALX4*	−2.34	2.1 × 10^–2^	0.0019	0.0021	0.0025	0.0023	3.8 × 10^–1^
*ALX1*	−2.89	2.9 × 10^–2^	0.0009	0.0022	0.0015	0.0042	1.2 × 10^–2^
*PVALB*	−3.71	1.6 × 10^–2^	0.0350	0.3322	0.0942	0.3272	6.6 × 10^–8^

*logFC* Logarithm of Fold Change, *P*_*adj*_ adjusted *p*-value

### DNA methylation differences

**Fig. 4:**
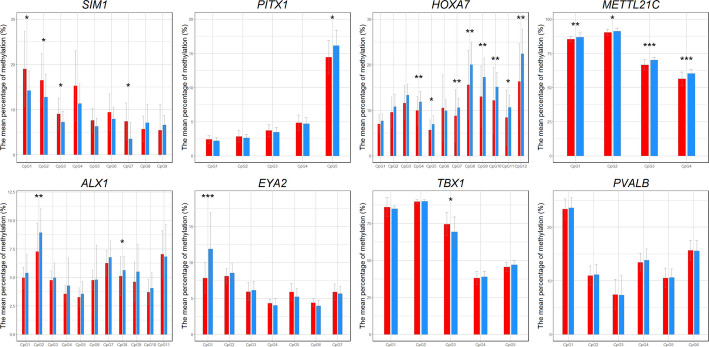
Mean percentage of DNA methylation ± SD in the CpG of *SIM1*, *PITX1*, *HOXA7*, *METTL21C*, *PVALB*, *ALX1*, *EYA2*, and *TBX1* for two groups: UH (red bars) and the control group (blue bars). Asterisks indicate statistical significances between the UH and control groups: one asterisk (*) denotes a *p*-value less than 0.05, two asterisks (**) denote a *p*-value less than 0.01, and three asterisks (***) denote a *p*-value less than 0.001

For eight significant genes that passed qPCR validation, the CpG methylation level was determined in the sequence fragments. The number of cytosines examined in the context of CpG varied depended on the gene. Detailed results, with the location of the cytosines in the genome, are shown in Additional File 1, Table S3 and Fig. 4. The methylation of cytosines decreased for *PITX1*, *HOXA7*, and *METTL21C*, which corresponded with observed higher expression. The significance test for Spearman’s correlation coefficient yielded significant negative correlation (decreased methylation accompanied by increased expression) for three out of the four analyzed cytosines in *METTL21C* at significance level 0.01 and for eight CpG sites in *HOXA7* at significance level 0.01 with the highest effect for CpG12 ($\rho =$ −0.7365; *p* < 0.0001). No significant correlation between methylation level and qPCR result was observed for the only differentially methylated CpG site in *PITX1* gene (Additional File 1, Table S3). For *EYA2* and *ALX1*, lower levels of methylation were observed. However, these genes were downregulated in the RNA-seq analysis. No significant correlation was observed between methylation level and qPCR outcomes for these genes. A higher level of methylation was observed for *SIM1* and *TBX1*; however, this is in agreement only for *TBX1*, where transcription was downregulated, while in *SIM1* it was upregulated. The significance test for Spearman’s correlation coefficient revealed one significant negative correlation for CpG in *TBX1* (*p* < 0.001) and a significant positive correlation for four differentially methylated cytosines in *SIM1* at significance level 0.01 (Additional File 1, Table S3).

### DNA variants in 5’-flanking regions

DNA sequencing analysis revealed that the number of known SNP variants varied from none for the *ALX1* gene to five for the *EYA2* gene. The genotype frequencies, as well as the allele frequencies for all identified SNPs, are shown in Table 4. We compared the allele frequencies of animals that developed UH (n = 34) to those in the control group (*n* = 34) using the Odds ratio test.

**Table 4:** Allele frequencies with odds ratio

Gene	Variant (rs ID) and genomic localization	Allele	Frequency		*P*-value	Odds ratio
			**UH**	**C**		
*EYA2*	rs3473097292 Ref:A > Alt:G SSC17: 48,966,057 bp	A	0.34	0.35	0.8569	1.0672
		G	0.66	0.65		
	rs3469840384 Ref:G > Alt:T SSC17: 48,966,127 bp	G	0.34	0.35	0.8569	1.0672
		T	0.66	0.65		
	rs3474717637 Ref:T > Alt:C SSC17: 48,966,165 bp	T	0.34	0.35	0.8569	1.0672
		C	0.66	0.65		
	rs3471363061 Ref:G > Alt:A SSC17: 48,966,249 bp	G	0.96	0.96	1	1
		A	0.04	0.04		
	rs3475300388 Ref:A > Alt:G SSC17: 48,966,463 bp	A	0.96	0.96	1	1
		G	0.04	0.04		
*HOXA7*	rs345656048 Ref:G > Alt:T SSC18: 45,416,713 bp	G	0.41	0.43	0.8620	1.0623
		T	0.59	0.57		
*METTL21C*	rs330073569 Ref:C > Alt:T SSC11: 71,021,084 bp	C	0.57	0.75	0.0311^*^	2.2308
		T	0.43	0.25		
	rs342909836 Ref:G > Alt:A SSC11: 71,021,427 bp	G	0.97	1	0.2930	5.1504
		A	0.03	0		
	rs1110951550 Ref:CTT > Alt:- SSC11: 71,021,062 bp—SSC11: 71,021,064 bp	CTT	0.78	0.81	0.6396	1.2204
		Del	0.22	0.19		
*PITX1*	rs3471329176 Ref:G > Alt:A SSC2: 137,207,515 bp	G	0.66	0.66	1	1
		A	0.34	0.34		
	rs3474364796 Ref:A > Alt:G SSC2: 137,207,408 bp	A	0.44	0.44	1	1
		G	0.56	0.56		
	rs3470743583 Ref:C > Alt:T SSC2: 137,207,386 bp	C	0.66	0.68	0.8554	1.0687
		T	0.34	0.32		
*PVALB*	rs340967655 Ref:G > Alt:A SSC5: 10,955,383 bp	G	0.84	0.71	0.0691	0.4632
		A	0.16	0.29		
*SIM1*	rs336725934 Ref:C > Alt:G SSC1: 67,294,204 bp	C	0.54	0.53	0.3915	1.3427
		G	0.46	0.47		
*TBX1*	rs331402937 Ref:C > Alt:T SSC14: 51,289,309 bp	C	0.62	0.74	0.1443	1.7196
		T	0.38	0.26		

*Ref* Reference, *Alt* Alternative;

^*^Significant* p*-value

**Fig. 5:**
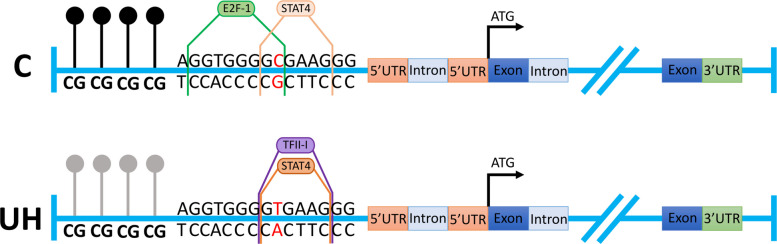
Sequence fragment of *METTL21C* potential promotor region with found SNP variant, transcriptional factors binding sites, transcriptional factors, and schematic presentation of lower (gray circles) methylation level of four cytosines in UH cases versus controls (black circles)

The most interesting result was for the *METTL21C* gene, where the SNP rs330073569 showed a significant association (*p* = 0.03) with a higher frequency of allele T in UH pigs (0.43) than in control pigs (0.25) (Table 4). Additionally, in silico analysis of the 5’-flanking region of this gene revealed that the identified variant was located within the consensus sequence for the E2F-1 and STAT4 transcription factors (Fig. 5, Additional File 1, Table S5). In the case of the T allele, this variant leads for loss of the E2F-1 binding site, for a greater similarity to the studied region for STAT4, and for the occurrence of a binding site for the TFII-I transcription factor. Additionally, within the region where the identified SNP was found, the NNPP tool showed a promoter-like sequence with a score of 0.98. Data from The Pig RNA Atlas confirmed high baseline expression of *METTL21C* gene in porcine skeletal muscle, however this SNP is not recorded in this database.

No significant results in terms of allele frequencies were observed for the *SIM1*, *PITX1*, *HOXA7*, *PVALB*, *ALX1*, *EYA2*, and *TBX1* genes.

### Screening for variants associated with umbilical hernia

The analysis of RNA-seq results identified 92,955 SNPs in the transcribed regions, with 1,369 variants significant in their frequencies (*p* < 0.05) according to the Fisher test. Among the identified polymorphisms, 80 were missense variants, 54 were 5’UTR variants, and 381 were located in 3’UTR region (Additional File 4). Based on the significant *p*-value and the logFC score from the RNA-seq data, as well as on the function of the particular genes, we selected four variants for further analysis in the larger cohort of 34 UH and 34 control pigs. These were: rs341151132 in *GALNT16*, rs690269483 in *PAOX*, rs334837422 in *RFTN1*, and rs318378401 in *SLC9A1*. The allele frequencies were calculated and the two-tailed Fisher’s exact test showed that there were no significant results in terms of allele frequencies for the examined variants (Additional File 1, Table S6).

### Copy number variation

We used droplet digital PCR (ddPCR) analysis to determine whether the differences in the mRNA level could be caused by CNV polymorphism affecting the studied genes. No instances of CNV exceeding two copies per fragment were detected in either the case or the control groups for any of the studied genes (Additional File 1, Figure S1).

### Protein level

Western blot analysis was performed for four selected proteins: SIM1, PITX1, EYA2, and TBX1. In case of EYA2 and TBX1, two isoforms were observed for both proteins (60 kDa and 55 kDa for EYA2, 60 kDa and 50 kDa for TBX1). For the remaining proteins, the specific bands were as follows: 70 kDa for SIM1 and 40 kDa for PITX1 (Additional File 1, Figure S2, Additional File 1, Figure S3). Statistical analysis of all the proteins and isoforms showed no significant results when comparing UH animals with control animals.

## Discussion

Understanding the genetic background of many diseases is extremely important, as the alteration of the gene function can directly lead to the development of certain anomalies, such as umbilical hernia. Our knowledge of the genes involved in the formation of UH is still limited and, despite the importance of this issue in pig farming, research on this topic remains sparse. Previous studies have used various methodologies and have identified some potential genes and significant biological processes associated with umbilical hernia in pigs. Those investigations were focused on uncovering the genetic factors that contribute to UH, including identification of SNPs, QTL regions, and changes in genes expression [1, 3, 4, 9, 13, 14, 35], however our knowledge of UH in pigs is still insufficient for a full understanding of the origins of this disorder.

Here we used a case–control study based on RNA-seq analysis to identify genes with alternated transcript levels that were associated with the occurrence of umbilical hernia in pigs. Through a multifaceted approach involving transcriptomics, epigenetics, and genomics, we identified differentially expressed genes and associated pathways, and also explored potential genetic variants and epigenetic modifications contributing to the occurrence of the observed changes in expression levels. To our knowledge, this is the first study to conduct an in-depth transcriptome analysis specifically on muscle tissue from UH and control animals. Focusing on this tissue provides valuable information on molecular changes that are specific to muscle. This tissue-specific approach helps to better understand the biological processes and pathways involved in muscle growth and maintenance, the proper functioning of which may be important in the context of hernia pathogenesis. The advantage of analyzing muscle tissue is the ability to directly investigate alterations in genes involved in muscle development, structure, and repair, which may be central to UH pathogenesis. In contrast, studies based on umbilical cord or heterogeneous tissue samples may dilute muscle-specific signals, potentially obscuring relevant biological processes. A potential limitation of focusing on a single tissue type is possibility to miss other factors such as extracellular matrix remodeling or vascular development, which could be captured in more complex tissue samples.

Our global transcript level analysis found 59 significant DEGs between UH and control animals, with a balanced distribution of upregulated and downregulated genes. The criteria employed (FDR < 0.05 and $\left|{\text{log}}_{2}FC\right|>1$) ensured the robustness and reliability of these findings. qPCR validation confirmed the RNA-seq results (with a high correlation), demonstrating consistent differential expression for most of the selected genes, with eight of eleven retaining statistical significance—namely, *SIM1*, *PITX1*, *HOXA7*, *METTL21C*, *PVALB*, *ALX1*, *EYA2*, and *TBX1.*

The functional enrichment analysis further underscored the biological relevance of these DEGs, identifying significant GO terms associated with critical pathways. Notably, the analysis identified sixty significant biological processes and sixteen significant molecular functions. These GO terms are associated with critical cellular functions and pathways, emphasizing the differential gene expression patterns between UH and control conditions. The GO analysis revealed the significant involvement of genes associated with processes potentially associated with the development of umbilical hernia such as related to skeletal system development and skeletal morphogenesis (GO:0001501, GO:0048704 and GO:0048705). It is understood that skeletal development encompasses muscle formation, with both tissues developing in a coordinated manner [36]. Moreover, some researchers have proposed that bone tissue can affect skeletal muscle metabolism, either directly or indirectly. These results suggest that disruptions to these pathways could contribute to the weakening of muscle structure and connective tissue around the navel [37]. This, in turn, could predispose to the development of structural malfunctions such as umbilical hernias. Notably, among the genes identified within these GO terms, *ALX1* and *HOXA7* were statistically significant in qPCR validation conducted on a larger pool of samples. While the connection between *HOXA7* and muscle tissue remains less well-defined, it is recognized that Hox genes, in general, are crucial in the development and patterning of muscle types across species, including *Drosophila* and vertebrates. Although the precise mechanisms remain unclear, numerous studies suggest that Hox genes, including *HOXA7*, may play roles in multiple stages of muscle development [38].

Other important GO pathways potentially associated with the development of umbilical hernia include processes associated with the development of embryonic organs and morphogenesis (GO:0048568, GO:0048598, GO:0048562, GO:0009790, GO:0043009), in which *HOXA7* and *ALX1* genes are also involved, along with *TBX1*. During development, certain defects or malformations can potentially lead to hernia formation. In particular, due to a complex structure that forms during embryonic development, the umbilicus remains a weak point in the body structure which is associated with the risk of hernia in later life [39].

In order to find factors responsible for the observed differences in the expression of eight statistically significant genes, we conducted a detailed analysis of DNA methylation patterns, while also seeking SNPs and CNVs.

DNA methylation, particularly in regulatory regions, is a crucial epigenetic modification that regulates gene expression by influencing the accessibility of transcription factors to the DNA. We observed hypomethylation at one specific CpG site of *PITX1* and an upregulation of this gene in UH pigs. However, no significant correlation was found between these variables, indicating that other regulatory mechanisms may be involved. This gene is known for its role in lower limb development, where it regulates the expression of genes responsible for muscle development. Dysfunction in *PITX1* can lead to abnormalities in muscle structure [40, 41]. We also observed extensive hypomethylation of the 5’-flanking region of the *HOXA7* gene with significant negative correlation with its increased expression in UH animals. These findings may contribute to the altered developmental processes observed in UH. For *METTL21C*, four significant CpG sites showed decreased methylation in the UH group and three of them showed a significant negative correlation with qPCR results. This hypomethylation aligns with the observed upregulation of *METTL21C* in UH pigs. *METTL21C* is involved in the methylation of lysine residues on proteins, a process that can affect protein function and stability [42], particularly in muscle tissue [43]. The relation between hypomethylation and increased *METTL21C* expression suggests that epigenetic regulation may play an important role in the gene’s contribution to UH. One hypermethylated CpG was found in 5’UTR region of *TBX1*, a gene belonging to the TBX transcription factor family that contains over twenty essential genes involved in embryonic development [44, 45]. The *TBX1* gene is responsible for the regulation of myocyte number and muscle development [44]. Some studies have previously indicated a potential association of *TBX1* with congenital diaphragmatic hernia [46] and inguinal hernia in humans [47]. In that study, a polymorphism was found in the promoter region, which can alter *TBX1* levels [47], while in our study the only identified SNP variant (rs331402937) in the 5’-flanking region did not differ in allele frequency between UH and control groups. Our finding of hypermethylated cytosine of *TBX1* corresponds with the observed decreased transcript level of this gene (with significant negative correlation). Some studies have suggested that methylation in the 5’UTR may alter the binding sites of regulatory proteins that influence gene expression, as is the case with the promoter [48]. These results may point to an important role for *TBX1* in the development of hernia in pigs. Interestingly, for *SIM1*—despite the hypermethylation observed in the 5’-flanking region, with significant correlation—the gene was upregulated in UH pigs. *SIM1* gene is primarily known for its role in the development of the central nervous system. However, evidence suggests that *Sim* genes may also be crucial in limb muscle formation in vertebrates, with studies confirming the association of *SIM1* expression with muscle progenitor cell migration in mice and chickens [49]. For *EYA2*, seven CpG sites were examined, with one showing a significant decrease in methylation in UH pigs. This gene plays essential roles in the specification of hypaxial muscle and in guiding the migration of hypaxial myogenic precursor cells [50]. Genes of the *EYA* family are particularly significant in the development of the eyes, heart, kidneys, and muscles and are also involved in the development of various organs during embryogenesis; their mutations can lead to congenital defects. Despite the fact that *EYA2* is rarely linked to disorders [51], in this study the *EYA2* mRNA level was downregulated in UH pigs, but similarly to *SIM1*, DNA methylation probably is not crucial for its expression. In case of the *ALX1* gene, two CpG sites had significantly lower methylation level. However, the Spearman's rank correlation coefficient demonstrated an absence of significant correlation with the results obtained from qPCR. This gene plays a crucial role in the development of craniofacial structures and is associated with various congenital defects, particularly those affecting the face. Mutations in *ALX1* are linked to frontonasal dysplasia syndrome [52]. As with *EYA2* and *SIM1*, expression of *ALX1* was downregulated while hypomethylation of the 5’-flanking region was observed. This unexpected outcome observed for *SIM1*, *EYA2*, and *ALX1*—in which DNA methylation within regulatory regions correlates positively with gene expression—may point to complex epigenetic regulation, where methylation does not act solely as a repressive mark. One explanation is that methylation prevents the binding of transcriptional repressors rather than activators, thereby promoting gene expression. Alternatively, methylation in non-CpG island promoters or in enhancer-like regulatory regions may stabilize open chromatin conformations or facilitate the recruitment of methyl-binding proteins (MeCP2 or MBD2) that act as transcriptional co-activators [53]. These observations emphasize the idea that the regulatory role of methylation is highly context-dependent, and influenced by local chromatin environment, transcription factor availability, and three-dimensional genome organization [54–56]. Moreover, numerous transcription factor binding events have been identified within the 5'UTR region of the human *SIM1* gene based on ChIP-seq data. This region also contains CpG islands, whose methylation may affect the accessibility of transcription factor binding sites. According to ChIP-seq data (GSE76494) [57], binding of the SP1 was confirmed in the 5'UTR region of *SIM1*, although the analysis was performed in HEK293 cells. The presence of SP1 in this region, combined with its known ability to bind methylated CpG islands [58], suggests a potential mechanism for maintaining *SIM1* expression despite promoter hypermethylation. Although this experiment was conducted in non-muscle cells, considering the broad expression of SP1 and including its high expression in skeletal muscle (The Pig RNA Atlas, The Human Protein Atlas), SP1 role in regulating *SIM1* expression in pigs should be further explored. For the *PVALB* gene, which has been associated with muscle growth in pigs [59], no significant in methylation level cytosines were observed, despite six of them being analysed.

Our study also identified SNPs within the 5’-flanking regions of several DEGs, but only one for the *METTL21C* gene was statistically significant. SNP rs330073569 showed a higher frequency of the T allele in UH animals, which could potentially affect transcription factor binding and gene expression. In silico analysis showed that the polymorphism results in a significant alteration of transcription factor binding dynamics. In the C variant, both STAT4 and E2F-1 can bind to this site, with a dissimilarity score of 4.41% for STAT4. However, the occurrence of a T variant leads to the loss of E2F-1 binding, with a reduced dissimilarity score of 2.94% for STAT4, and also introduces a new binding site for TFII-I. This change in transcription factor dynamics could have important implications for the gene’s regulation and function, potentially contributing to the development of UH as we observe an increased level of *METTL21C* transcript. STAT4 is transcription activator and is known to play roles in immune responses and cell proliferation [60]. Its greater similarity to the studied regulatory region may be the cause of the observed stronger transcriptional activation effect. Additionally, the introduction of TFII-I, which can act as a transcriptional coactivator [61], might further enhance *METTL21C* transcription in the occurrence of the T variant. It was already shown that the E2F-1 transcription factor can act as repressor of transcription [62, 63], thus loss of E2F-1 binding to the studied region in the case of the T allele might lead to increased transcription of the *METTL21C* gene. Another explanation is that, despite the lack of E2F-1, the presence of other transcription factors (such as STAT4 and TFII-I) can compensate for or even enhance transcriptional activity. *METTL21* gene encodes nonhistone methyltransferase, an enzyme from the *METTL* subfamily which is responsible for methylation of nonhistone lysine. It has been demonstrated that disruption of the function of the *METTL21C* gene can lead to significant impairments in muscle function. *METTL21C* plays a crucial role in methylating specific proteins, such as p97, which is involved in protein degradation processes like autophagy and the ubiquitin–proteasome system. If *METTL21C* is disrupted, it can lead to impaired methylation of p97, which then affects its ability to form hexamers and carry out its function in removing damaged or misfolded proteins from muscle cells [43]. Studies also suggest that, in trimethylating Hspa8/HSC70, *METTL21C* is particularly important for slow muscle fibers, which are vital for endurance and sustained muscle activity [64]. Analysis of publicly available datasets (The Pig RNA Atlas) confirms that *METTL21C* shows tissue-enhanced expression in porcine skeletal muscle, with markedly higher transcript levels compared to other tissues. Similar patterns are observed in human data, where *METTL21C* is also group-enriched in skeletal muscle and epididymis. These findings are consistent with our own results showing upregulated *METTL21C* expression in muscle tissue from umbilical hernia-affected pigs, further supporting the biological relevance of this gene in abdominal muscle structure and integrity. However, the identified here SNP (rs330073569) is not recorded in this database, but neighboring variant (rs341325198) located 664 bp upstream from our SNP is indicated as an expression Quantitative Trait Loci (eQTL) in muscle tissue. Thus, we can suspect that also rs330073569 located in potential promoter region (354 bp from exon 1) should be considered as potential cis-regulator (moreover, that we show that the presence of this SNP changes the sites for potential transcription factors). Moreover, both hypomethylation and rs330073569 were identified in the upstream region of the *METTL21C* gene. These two regulatory changes may independently or interactively influence gene expression. Hypomethylation of promoter CpG sites is typically associated with increased transcriptional activity, as observed here with upregulated *METTL21C* expression. Simultaneously, the T allele of rs330073569 alters predicted transcription factor binding sites. Although our study did not experimentally test the interplay between SNP and methylation effects, the co-occurrence of both changes in UH pigs suggests that they may act synergistically to enhance *METTL21C* transcription. Hypomethylation could increase chromatin accessibility [65], thereby facilitating binding of TFs favored by the T allele configuration. Conversely, it is also possible that the presence of the SNP alters local chromatin dynamics [66], indirectly influencing methylation susceptibility. Future functional studies will be necessary to dissect the individual and combined effects of these regulatory elements. Considering the above and the importance of this gene, we hypothesize that the disruption of *METTL21C* gene expression observed here can lead to the impaired methylation of other proteins, resulting in malfunction and their contribution to muscle dysfunction.

The ddPCR analysis conducted in this study found no evidence of CNV exceeding the normal two copies per gene in either UH or control pigs. This finding suggests that CNVs are not a cause of altered transcript level of these genes. However, it should be pointed out that certain CNVs, particularly CNV SSC14: 13,030,843 bp – SSC13059455 bp, showed a significant association with UH in Duroc pigs, specifically affecting the *NUGGC* gene, which is known to be implicated in human omphalocele and inguinal hernia. This rare CNV was found exclusively in the Duroc breed [14]. The absence of CNV alterations in the studied genes in our population suggest that the occurrence of UH may be more strongly affected by changes in gene regulation rather than by gene dosage effects. This conclusion directs future research towards investigations of regulatory mechanisms, such as transcription factor activity or post-transcriptional modifications, which might better explain the molecular basis of UH.

The western blot analysis was performed for a limited number of proteins (*SIM1*, *PITX1*, *EYA2* and *TBX1*) on account of antibody-specific problems for the pig. The analysis of protein levels did not reveal significant differences between the UH and control animals. This could be explained by posttranscriptional regulatory mechanisms, to protein stability, or to experimental limitations, as it was performed only on a small animal cohort (*n* = 12 per group). As previously reviewed by Wu & Brewer [67], mRNA stability is an essential posttranscriptional regulatory process that can affect gene expression without corresponding changes in protein production. The half-life of mRNA, regulated by RNA-binding proteins and microRNAs, can affect transcript abundance and persistence in the cell. Future studies employing more sensitive and quantitative proteomic approaches might be necessary to elucidate the protein-level alterations associated with UH.

The comprehensive RNA-seq analysis identified a substantial number of SNPs within the transcribed regions, highlighting potential genetic variability linked to umbilical hernia.

On the basis of their traits, such as *p*-value or biological function, four candidate SNPs (rs341151132 in *GALNT16*, rs690269483 in *PAOX*, rs334837422 in *RFTN1*, and rs318378401 in *SLC9A1*) were selected for analysis in a larger group of animals. *GALNT16* is an enzyme that belongs to a family responsible for the process of O-glycosylation and is involved in many processes such as metabolism of proteins and lipids, prolactin, and AMPK signaling pathways, as well as the insulin/IGF pathway involving the protein kinase B signaling cascade [68]. Although *GALNT16* has not been directly associated with muscle function, the process of glycosylation is essential for proper muscle physiology. In fact, defects in protein glycosylation have been linked to several types of congenital muscular dystrophy [69]. *RFTN1* is involved in immune cell signaling and in maintaining lipid rafts, which are critical for membrane organization in cells, including in immune cells [70]. There is no evidence that this gene directly affects muscle tissue. However, since *RFTN1* plays a role in immune cell signaling, it may hypothetically affect muscle function in the context of autoimmune diseases, where the immune system mistakenly attacks muscle tissue, as in myositis. *PAOX* encodes enzyme acetylpolyamine oxidase, which is involved in the catabolism of spermine [71]. These discoveries suggest the important role of this protein in muscle, as accumulation of this polyamine has been observed in hypertrophy and decreased levels have been noted in atrophy [72]. The *SLC9A1* gene is responsible for the regulation of intracellular pH. A decrease in its expression can potentially lead to acidification, which may in turn lead to changes in myofilament sensitivity to Ca^2+^, as has been noted in ventricular myocytes of aging Goto Kakizaki rats and diabetic rats induced by streptozotocin [73]. Despite the occurrence of significant SNPs in RNA-seq analysis, the Sanger sequencing in the larger cohort (n = 34 per group) did not find any significant differences in allele frequencies between the UH and control groups.

While this study provides another element that brings us closer to a better understanding of hernias background, several limitations should be acknowledged. The sample size, although adequate for initial discovery, may limit the generalizability of the findings. Moreover, our previous studies have shown that the frequency of polymorphisms varies in cohorts of animals containing different gene pools, and also that some prior findings have not been confirmed in other breeds [16, 17]. Larger cohorts, independent validation, and studies on different breeds are therefore required to confirm these results. An unexpected clustering of three samples observed in the hierarchical heatmap could be explained by biological variability or technical noise. qPCR validation of key transcripts showed no deviation from group means, suggesting that the clustering discrepancy does not indicate systematic differences in expression. Additionally, functional studies are needed to elucidate the precise roles of the identified genes and pathways in hernia development and to explore potential interactions between genetic, epigenetic, and environmental factors in UH.

Summarizing, we observed indications of consistent patterns of dysregulation across multiple molecular layers in muscle tissue from pigs affected by umbilical hernia (UH). *METTL21C* emerged as the most prominent candidate, showing a strong convergence of molecular signals, including elevated transcript levels, decreased promoter methylation, and the presence of a variant (rs330073569) within a predicted transcription factor binding site. Several other genes such as *SIM1*, *PITX1*, *HOXA7*, *PVALB*, *ALX4*, *EYA2*, and *TBX1* also exhibited significant changes in transcript level, confirmed by both RNA-seq and qPCR analyses. Moreover, for *PITX1*, *HOXA7* and *TBX1*, transcript abundance was significantly correlated with promoter methylation status, suggesting a potential regulatory relationship. No copy number variations (CNVs) were identified and differences in protein levels did not reach significance.

## Conclusion

Our comprehensive analysis has identified significant genetic and epigenetic markers associated with umbilical hernia in pigs. These findings contribute to a better understanding of the complex biology underlying UH pathology. The innovative nature of our study, focusing on muscle tissue, extends existing knowledge on genetic background of UH.

We demonstrated for the first time the influence of an epigenetic mechanism (DNA methylation) on the regulation of mRNA levels of differentially expressed genes in pigs with umbilical hernia. Among the identified genes, *METTL21C* stands out as a particularly promising candidate due to its significant differential expression, partially caused by epigenetic regulation, and specific genetic variant within potential regulatory region.

## Supplementary Information

Supplementary Material 1.

Supplementary Material 2.

## Data Availability

The datasets used during this study are included in this published article or available from the corresponding author on reasonable request. The sequencing data have been submitted to the NCBI Sequence Read Archive (SRA) (accession number: PRJNA1147879).

## Abbreviations

Alt: Alternative
ALX1: ALX Homeobox 1
ALX4: ALX Homeobox 4
BWD: Body wall defect
C: Control
CPM: Counts per million
CNV: Copy number variation
ddPCR: Droplet digital PCR
DEG: Differentially expressed gene
E2F-1: E2F Transcription Factor 1
EYA2: EYA Transcriptional coactivator and phosphatase 2
F2: Coagulation factor II gene
FAM: Fluorescein
FM: Female
FDR: False discovery rate
GALNT16: Polypeptide N-Acetylgalactosaminyltransferase 16
GAPDH: Glyceraldehyde-3-phosphate dehydrogenase
GATK: The Genome Analysis Toolkit
GO: Gene ontology
H3F3A: H3 histone, family 3A
HRP: Horseradish peroxidase
HEX: Hexachlorofluorescein
HOXA7: Homeobox A7
IRX5: Iroquois Homeobox 5
ITGAM: Integrin α M
logFC: Logarithm of Fold Change
M: Male
Mcl-1: MCL1 Apoptosis Regulator, BCL2 family member
METTL21C: Methyltransferase 21C
MMP13: Matrix Metallopeptidase 13
NCBI: National Center for Biotechnology Information
OSM: Oncostatin M
OSR2: Odd-Skipped Related Transcription Factor 2
P_adj_: Adjusted *p*-value
PAOX: Polyamine Oxidase
PCA: Principal Component Analysis
PITX1: Paired Like Homeodomain 1
PPIA: Peptidyl-prolyl cis–trans isomerase A
PVALB: Parvalbumin
Ref: Reference
qPCR: Quantitative polymerase chain reaction
QTL: Quantitative Trait Loci
RFTN1: Raftlin, Lipid Raft Linker 1
RIN: RNA integrity numbers
SIM1: SIM bHLH transcription factor 1
SLC9A1: Solute Carrier Family 9 Member A1
SNP: Single nucleotide polymorphism
SRA: NCBI Sequence Read Archive
STAR: Spliced Transcripts Alignment to a Reference
STAT4: Signal Transducer and Activator of Transcription 4
TBX1: T-Box Transcription Factor 1
TFII-I: General Transcription Factor IIi
TMM: Trimmed Mean of M-values
UH: Umbilical hernia
VEP: Variant Effect Predictor
