# Supplementary material for: Genetic and epigenetic markers in the METTL21C gene associated with umbilical hernia in pigs
Source: BMC Genomics. 2025 Nov 18;26:1096. doi: 10.1186/s12864-025-12315-0 (PMC12696900; doi:10.1186/s12864-025-12315-0)
Supplement: Supplementary file 1 — Supplementary Material 1. [file 12864_2025_12315_MOESM1_ESM.docx]

**Additional file 1 for:**

**Genetic and epigenetic markers in the *METTL21C* gene associated with umbilical hernia in pigs** by Wozniak et al.

Table S1. Summary of animals included in the study and the types of analysis performed.

| **Animals** | **Sex** | **Mother’s identifier number** | **Type of analysis** | | | | | | |
| --- | --- | --- | --- | --- | --- | --- | --- | --- | --- |
|  |  |  | **RNA-seq** | **qPCR** | **Pyrosequencing** | **Sanger sequencing of promotor region** | **Sanger sequencing of RNA-seq indicated variants** | **ddPCR of CNVs** | **Western blot** |
| C1 | FM | 1 |  | ✓ | ✓ | ✓ | ✓ | ✓ | ✓ |
| C2 | FM | 1 |  | ✓ | ✓ | ✓ | ✓ | ✓ | ✓ |
| C3 | M | 2 | ✓ | ✓ | ✓ | ✓ | ✓ | ✓ | ✓ |
| C4 | FM | 2 |  | ✓ | ✓ | ✓ | ✓ | ✓ | ✓ |
| C5 | M | 4 | ✓ | ✓ | ✓ | ✓ | ✓ | ✓ | ✓ |
| C6 | FM | 5 |  | ✓ | ✓ | ✓ | ✓ | ✓ | ✓ |
| C7 | M | 6 | ✓ | ✓ | ✓ | ✓ | ✓ | ✓ | ✓ |
| C8 | M | 6 |  | ✓ | ✓ | ✓ | ✓ | ✓ | ✓ |
| C9 | FM | 7 |  | ✓ | ✓ | ✓ | ✓ | ✓ | ✓ |
| C10 | FM | 7 |  | ✓ | ✓ | ✓ | ✓ | ✓ | ✓ |
| C11 | FM | 8 |  | ✓ | ✓ | ✓ | ✓ | ✓ | ✓ |
| C12 | FM | 8 |  | ✓ | ✓ | ✓ | ✓ | ✓ | ✓ |
| C13 | M | 9 |  | ✓ | ✓ | ✓ | ✓ | ✓ |  |
| C14 | FM | 10 |  | ✓ | ✓ | ✓ | ✓ | ✓ |  |
| C15 | FM | 11 |  | ✓ | ✓ | ✓ | ✓ | ✓ |  |
| C16 | FM | 12 | ✓ | ✓ | ✓ | ✓ | ✓ | ✓ |  |
| C17 | FM | 13 | ✓ | ✓ | ✓ | ✓ | ✓ | ✓ |  |
| C18 | FM | 13 | ✓ | ✓ | ✓ | ✓ | ✓ | ✓ |  |
| C19 | M | 14 | ✓ | ✓ | ✓ | ✓ | ✓ | ✓ |  |
| C20 | FM | 15 | ✓ | ✓ | ✓ | ✓ | ✓ | ✓ |  |
| C21 | FM | 16 | ✓ | ✓ | ✓ | ✓ | ✓ | ✓ |  |
| C22 | M | 17 | ✓ | ✓ | ✓ | ✓ | ✓ | ✓ |  |
| C23 | M | 18 | ✓ | ✓ | ✓ | ✓ | ✓ | ✓ |  |
| C24 | FM | 19 | ✓ | ✓ | ✓ | ✓ | ✓ | ✓ |  |
| C25 | FM | 20 | ✓ | ✓ | ✓ | ✓ | ✓ | ✓ |  |
| C26 | FM | 21 | ✓ | ✓ | ✓ | ✓ | ✓ | ✓ |  |
| C27 | FM | 22 |  | ✓ | ✓ | ✓ | ✓ | ✓ |  |
| C28 | FM | 22 |  | ✓ | ✓ | ✓ | ✓ | ✓ |  |
| C30 | FM | 23 |  | ✓ | ✓ | ✓ | ✓ | ✓ |  |
| C31 | FM | 24 |  | ✓ | ✓ | ✓ | ✓ | ✓ |  |
| C32 | FM | 25 |  | ✓ | ✓ | ✓ | ✓ | ✓ |  |
| C33 | FM | 26 |  | ✓ | ✓ | ✓ | ✓ | ✓ |  |
| C34 | FM | 27 |  | ✓ | ✓ | ✓ | ✓ | ✓ |  |
| C35 | M | 28 | ✓ | ✓ | ✓ | ✓ | ✓ | ✓ |  |
| UH1 | FM | 1 |  | ✓ | ✓ | ✓ | ✓ | ✓ | ✓ |
| UH2 | FM | 1 |  | ✓ | ✓ | ✓ | ✓ | ✓ | ✓ |
| UH3 | M | 2 | ✓ | ✓ | ✓ | ✓ | ✓ | ✓ | ✓ |
| UH4 | FM | 2 |  | ✓ | ✓ | ✓ | ✓ | ✓ | ✓ |
| UH5 | M | 4 | ✓ | ✓ | ✓ | ✓ | ✓ | ✓ | ✓ |
| UH6 | FM | 5 |  | ✓ | ✓ | ✓ | ✓ | ✓ | ✓ |
| UH7 | M | 6 | ✓ | ✓ | ✓ | ✓ | ✓ | ✓ | ✓ |
| UH8 | M | 6 |  | ✓ | ✓ | ✓ | ✓ | ✓ | ✓ |
| UH9 | FM | 7 |  | ✓ | ✓ | ✓ | ✓ | ✓ | ✓ |
| UH10 | FM | 7 |  | ✓ | ✓ | ✓ | ✓ | ✓ | ✓ |
| UH11 | FM | 8 |  | ✓ | ✓ | ✓ | ✓ | ✓ | ✓ |
| UH12 | FM | 8 |  | ✓ | ✓ | ✓ | ✓ | ✓ | ✓ |
| UH13 | M | 9 |  | ✓ | ✓ | ✓ | ✓ | ✓ |  |
| UH14 | FM | 10 |  | ✓ | ✓ | ✓ | ✓ | ✓ |  |
| UH15 | FM | 11 |  | ✓ | ✓ | ✓ | ✓ | ✓ |  |
| UH16 | FM | 12 | ✓ | ✓ | ✓ | ✓ | ✓ | ✓ |  |
| UH17 | FM | 13 | ✓ | ✓ | ✓ | ✓ | ✓ | ✓ |  |
| UH18 | FM | 13 | ✓ | ✓ | ✓ | ✓ | ✓ | ✓ |  |
| UH19 | M | 14 | ✓ | ✓ | ✓ | ✓ | ✓ | ✓ |  |
| UH20 | FM | 15 | ✓ | ✓ | ✓ | ✓ | ✓ | ✓ |  |
| UH21 | FM | 16 | ✓ | ✓ | ✓ | ✓ | ✓ | ✓ |  |
| UH22 | M | 17 | ✓ | ✓ | ✓ | ✓ | ✓ | ✓ |  |
| UH23 | M | 18 | ✓ | ✓ | ✓ | ✓ | ✓ | ✓ |  |
| UH24 | FM | 19 | ✓ | ✓ | ✓ | ✓ | ✓ | ✓ |  |
| UH25 | FM | 20 | ✓ | ✓ | ✓ | ✓ | ✓ | ✓ |  |
| UH26 | FM | 21 | ✓ | ✓ | ✓ | ✓ | ✓ | ✓ |  |
| UH27 | FM | 22 |  | ✓ | ✓ | ✓ | ✓ | ✓ |  |
| UH28 | FM | 22 |  | ✓ | ✓ | ✓ | ✓ | ✓ |  |
| UH30 | FM | 23 |  | ✓ | ✓ | ✓ | ✓ | ✓ |  |
| UH31 | FM | 24 |  | ✓ | ✓ | ✓ | ✓ | ✓ |  |
| UH32 | FM | 25 |  | ✓ | ✓ | ✓ | ✓ | ✓ |  |
| UH33 | FM | 26 |  | ✓ | ✓ | ✓ | ✓ | ✓ |  |
| UH34 | FM | 27 |  | ✓ | ✓ | ✓ | ✓ | ✓ |  |
| UH35 | M | 28 | ✓ | ✓ | ✓ | ✓ | ✓ | ✓ |  |

M: Male; FM: Female; ✓: animal present in a particular analysis

Table S2. Primers and probes used in qPCR, Sanger sequencing, ddPCR, and pyrosequencing.

| **Target gene** | **Primer sequence 5’→ 3’** | **Amplicon size (bp)** | **Annealing temp. (^o^C)** |
| --- | --- | --- | --- |
| **Real-time PCR** | | | |
| *ALX1* | F: CTGGAGGAGCTGGAGAAGGT | 220 | 60 |
|  | R: TGTCAGTCCTGGGCAAAACT |  |  |
| *ALX4* | F: CACCCGAGCAGAGAACTACG | 173 | 56 |
|  | R: CCCCAGAGACACTCAGGAAG |  |  |
| *EYA2* | F: GTGTGTTCGTGTGGGACTTG | 148 | 60 |
|  | R: GTGTCTGCGAGGCTGAAGAT |  |  |
| *HOXA7* | F: AGGCCAATTTCCGCATTTAT | 124 | 58 |
|  | R: AGGTAGCGGTTGAAGTGGAA |  |  |
| *IRX5* | F: GCTACAACTCGCACCTCCAG | 124 | 60 |
|  | R: GGGTGGTACCCCAAGGAG |  |  |
| *METTL21C* | F:AGGAACATGCGGAGGAACTG | 312 | 60 |
|  | R:TGAGCAGCTTGTCCAGGAAG |  |  |
| *OSR2* | F:CCATCTCGGGCCTCAGTAAA | 319 | 60 |
|  | R:TGCATATGTAAAGTTTTGTGAACTGC |  |  |
| *PITX1* | F: GAGGAGATCGCTGTGTGGAC | 170 | 60 |
|  | R: GCGTACACATCCTCGTAGGG |  |  |
| *PVALB* | F: TGGTTGGCCTGAAGAAAAAG | 165 | 60 |
|  | R: CCATCAGCGTCTTGGTTTCT |  |  |
| *SIM1* | F: ATTGAGCGCTCCTTCTTCCT | 128 | 60 |
|  | R: ATGTCCAGGCTGTACTGTCG |  |  |
| *TBX1* | F:CCATGCACAGATACCAGCCA | 201 | 60 |
|  | R:GATCGCAATCTCGGAAGCCT |  |  |
| **Sanger sequencing analysis of promoter regions** | | | |
| *ALX1* | F: ACGAGCCTCCCTTTGGTCTA | 790 | 60 |
|  | R: TTGAAACTCAGCTGGGCTCC |  |  |
| *EYA2* | F: CATGTGGAGGGGCGTGTAC | 516 | 63 |
|  | R: CCCACACCTCCTCCCTCTAT |  |  |
| *HOXA7* | F: TAGAGGGCGGGTAAACAGTG | 761 | 60 |
|  | R: GCTAAAAAGCGCGTTCACAT |  |  |
| *METTL21C* | F:GCTCACTGGGTAAGCATGGT | 700 | 64 |
|  | R:TACCCACCAGTAGCCAGACA |  |  |
| *PITX1* | F:CAAGCTAATTAGGAGGCAGCA | 693 | 65 |
|  | R:GCCTGTCTTAAAGCGACAGC |  |  |
| *PVALB* | F:CAGATGTGGGATGGGGCAC | 260 | 64 |
|  | R:GCGCATCTCCTAGGATGTCC |  |  |
| *SIM1* | F:CTGGCCTATTCCTGCCTCTC | 774 | 62 |
|  | R:TCCGCTTCACCTACCTTCAG |  |  |
| *TBX1* | F:AGGCACTTTCCGCTGGAG | 600 | 60 |
|  | R:GGCCAGGACGCTTATACATC |  |  |
| **Sanger sequencing analysis of RNA-seq indicated variants** | | | |
| *GALNT16* | F:ACCTGTCTCATTCTGAATGAAGGA | 380 | 60 |
|  | R:TGGCTTCTCTGTTTCTAGGGC |  |  |
| *PAOX* | F:AGCCGTCATCACAACACAGT | 543 | 60 |
|  | R:CTTCTTGAACCAGGCGTCCT |  |  |
| *RFTN1* | F:CCCTCACAGATCCAGGAAGC | 532 | 60 |
|  | R:ACACGTGACCTTTTGGAGCA |  |  |
| *SLC9A1* | F:GCGTCTGTCCTTCACCCTAG | 217 | 60 |
|  | R:TCTTTTGTACAGGCGGCAGA |  |  |
| **Pyrosequencing analysis** | | | |
| *ALX1* | F:GGAGAGTGGAGGGTTAATTG | 183 | 56 |
|  | R:5'biot-CCCCCATCTTCCCCTCTT |  |  |
|  | S:GTGGAGGGTTAATTGT |  |  |
| *EYA2* | F:5'TTGGTGTTGTAGATGTTTTATAGATG | 234 | 56 |
|  | R:5'biot-ACTCTCTACCCCTACAACCCA |  |  |
|  | S:5'TAGAGGGAGGAGGTG |  |  |
| *HOXA7* | F:5'GGATTTGAGTTTTAAGTTGGTTAATG | 165 | 56 |
|  | R:5’biot-TCCCTACCCTTCTTTCTACCA |  |  |
|  | S:5'GGTTAATGGTTTATTGGAT |  |  |
| *METTL21C* | F:5'biot-TGTTAGTATTAGGGTTTAGGGTAGAGTT | 264 | 56 |
|  | R:5'ACACACACACCCCATTCT |  |  |
|  | S:5'ATACAAACTAACACACTATAT |  |  |
| *PITX1* | F:5'GAGGGGTTTGTAGGTTAGAT | 79 | 56 |
|  | R:5'biot-TCACCCTCCTTCCCC |  |  |
|  | S:5'GGGTTTGTAGGTTAGAT |  |  |
| *PVALB* | F:5'AGAATGGGAGTAGGGTTAAGG | 225 | 56 |
|  | R:5'biot-AAACCCCCAAAAAAAAACACCTCAAATTT |  |  |
|  | S:5'GGGGTTTTGTATTTTATTAGG |  |  |
| *SIM1* | F:5'TTGGGATTAATTTAAAAATAATTTTTTGG | 201 | 56 |
|  | R:5'biot-ACAAAAAAAACAAAAATAAACCAAAC |  |  |
|  | S:5'AAAGAGGAAATATAATTTAATA |  |  |
| *TBX1* | F:5'TGGATGGGAAGGTTATGGA | 181 | 56 |
|  | R:5'biot-ACTTACTACCCACCATACAAC |  |  |
|  | S:5'GGAGGTTTTAGGGGTA |  |  |
| **Droplet digital PCR analysis** | | | |
| *ALX1* | F: 5’ CTCAAGAGCCCACCGAGTAA | 108 | 58 |
|  | R: 5’ CGACGCTTTGCTGTAAAAGGA |  |  |
|  | S: 5’ FAM- GGAGGCGCGTTAGAGCACGT -BQH1 |  |  |
| *EYA2* | F: 5’ AGGAAATGCTAGAACTAGTGATCTCA | 120 | 58 |
|  | R: 5’ GCCTACCTTCGATGTCACTCAG |  |  |
|  | S: 5’ FAM- CTGTAAACAGCGACCGTCCCGG -BQH1 |  |  |
| *HOXA7* | F: 5’ GAGCCGACTTCTTGCTCCTT | 112 | 58 |
|  | R: 5’ AGAGGGGGCTGTTGACATTG |  |  |
|  | S: 5’ FAM-GCCTTCCCTTCGACCGTGCC-BQH1 |  |  |
| *METTL21C* | F: 5’ TGGCCAGCTCTCTCCTCC | 119 | 58 |
|  | R: 5’ CTTGTCCCCCTCTGCCCT |  |  |
|  | S: 5’ FAM- CAGCAGCCCCAGCACCTGG-BQH1 |  |  |
| *PITX1* | F: 5’ CATGAGTCTGGAGCGGCTG | 80 | 58 |
|  | R: 5’ CTAGGTGGAAGGCGGGCC |  |  |
|  | S: 5’ FAM- CGCCGCCGCACGACATGG-BQH1 |  |  |
| *PVALB* | F: 5’ GGACATCCTAGGAGATGCGC | 86 | 58 |
|  | R: 5’ GAGTTCTCCTGCCGTCAAGG |  |  |
|  | S: 5’ FAM- GGCCCTGCACCTCATCAGGC-BQH1 |  |  |
| *SIM1* | F: 5’ TGGTTTTGGATGAATCTGTGGA | 94 | 58 |
|  | R: 5’ CCTAGTCCGGGCAGCATTTT |  |  |
|  | S: 5’ FAM- CGCCGCCGCACGACATGG-BQH1 |  |  |
| *TBX1* | F: 5’ CTCCGACCTGGTGAAGCTTC | 80 | 58 |
|  | R: 5’ CTTCCATGTCCCTGGTGACG |  |  |
|  | S: 5’ FAM- CCGGCCGGGATGCACTTCAG-BQH1 |  |  |
| *F2* | F: 5’ CTGCCAGCGGGCTGGGAATA 3’ | 97 | 58 |
|  | R: 5’ GGAGTTGACTCTGGAATAAGAAATTG 3’ |  |  |
|  | 5’ HEX-CGCCCCCGCCCCCAGGGTCT-BQH1 3’ |  |  |

Table S3. CpG sites in the analyzed genes with their genomic positions, genetic context, and Spearman’s correlation coefficients between methylation levels of significant CpG sites and gene expression measured by qPCR.

| **Gene** | **Genomic location of analysed sequence and gene context** | **No of analysed cytosines** | **Chromosome position of analysed cytosines** | **mean % level in UH group** | **mean % level in C group** | **Differences in methylation: *p*-value** | **Direction of expression change (UH vs. C) in qPCR** | **Correlation between the methylation level of significant CpG and qPCR results** | |
| --- | --- | --- | --- | --- | --- | --- | --- | --- | --- |
|  |  |  |  |  |  |  |  | **rho** | ***p*-value** |
| *ALX1* | SSC5: 96785265 bp - SSC5: 96785446 bp  5’flanking | CpG1 | 5:96785422 | 4.9706 | 5.3824 | 2.9×10^-1^ | Down | NA | NA |
|  |  | CpG^†^ | 5:96785403 | 7.2647 | 8.9412 | 1.2×10^-3**^ |  | 0.0223 | 8.6×10^−1^ |
|  |  | CpG3 | 5:96785384 | 4.7647 | 4.9706 | 2.9×10^-1^ |  | NA | NA |
|  |  | CpG4 | 5:96785382 | 3.5588 | 4.2647 | 6.0×10^-1^ |  | NA | NA |
|  |  | CpG5 | 5:96785379 | 3.2647 | 3.5588 | 1.1×10^-1^ |  | NA | NA |
|  |  | CpG6 | 5:96785360 | 4.7647 | 4.7941 | 8.1×10^-1^ |  | NA | NA |
|  |  | CpG7 | 5:96785352 | 6.2647 | 6.7647 | 2.3×10^-1^ |  | NA | NA |
|  |  | CpG8^†^ | 5:96785333 | 5.1176 | 5.6176 | 4.9×10^-2*^ |  | -0.0152 | 9.0×10^−1^ |
|  |  | CpG9 | 5:96785298 | 4.6176 | 5.5000 | 1.9×10^-1^ |  | NA | NA |
|  |  | CpG10 | 5:96785296 | 3.7059 | 4.0588 | 4.8×10^-1^ |  | NA | NA |
|  |  | CpG11 | 5:96785287 | 7.0294 | 6.8235 | 7.3×10^-1^ |  | NA | NA |
| *EYA2* | SSC17: 48966512 bp - SSC17: 48966631 bp  5’flanking | CpG1^†^ | 17:48966545 | 7.8235 | 11.8824 | 8.3×10^-4***^ | Down | 0.0335 | 7.9×10^−1^ |
|  |  | CpG2 | 17:48966572 | 8.0882 | 8.5294 | 1.8×10^-1^ |  | NA | NA |
|  |  | CpG3 | 17:48966578 | 5.9412 | 6.1471 | 5.0×10^-1^ |  | NA | NA |
|  |  | CpG4 | 17:48966596 | 4.3235 | 4.0294 | 1.4×10^-1^ |  | NA | NA |
|  |  | CpG5 | 17:48966600 | 5.8824 | 5.2353 | 7.8×10^-2^ |  | NA | NA |
|  |  | CpG6 | 17:48966602 | 4.3824 | 3.9706 | 6.9×10^-2^ |  | NA | NA |
|  |  | CpG7 | 17:48966606 | 5.8824 | 5.6765 | 5.6×10^-1^ |  | NA | NA |
| *HOXA7* | SSC18: 45416762 bp - SSC18: 45416903 bp  5’flanking | CpG1 | 18:45416789 | 7.0294 | 7.6765 | 8.3×10^-2^ | Up | NA | NA |
|  |  | CpG2 | 18:45416803 | 9.6176 | 10.8235 | 9.7×10^-2^ |  | NA | NA |
|  |  | CpG3 | 18:45416814 | 11.6176 | 13.3235 | 5.4×10^-2^ |  | NA | NA |
|  |  | CpG4^†^ | 18:45416829 | 9.9412 | 11.8824 | 2.8×10^-3**^ |  | -0.5278 | 3.7×10^−6***^ |
|  |  | CpG5^†^ | 18:45416831 | 5.6765 | 6.9706 | 1.4×10^-2*^ |  | -0.4305 | 2.5×10^−4***^ |
|  |  | CpG6 | 18:45416843 | 10.5294 | 9.9118 | 4.9×10^-1^ |  | NA | NA |
|  |  | CpG7^†^ | 18:45416853 | 8.8235 | 10.5882 | 1.2×10^-3**^ |  | -0.5496 | 1.2×10^−6***^ |
|  |  | CpG8^†^ | 18:45416858 | 15.6176 | 20.0294 | 8.1×10^-3**^ |  | -0.6643 | 6.6×10^−10***^ |
|  |  | CpG9^†^ | 18:45416862 | 13.0294 | 17.2941 | 3.5×10^-3**^ |  | -0.6929 | 5.9×10^−11***^ |
|  |  | CpG10^†^ | 18:45416868 | 12.1765 | 15.1471 | 1.6×10^-3**^ |  | -0.6539 | 1.5×10^−9***^ |
|  |  | CpG11^†^ | 18:45416870 | 8.4412 | 10.6471 | 1.4×10^-3**^ |  | -0.6174 | 2.0×10^−8***^ |
|  |  | CpG12^†^ | 18:45416877 | 16.3529 | 22.4118 | 2.2×10^-3**^ |  | -0.7365 | 8.2×10^−13***^ |
| *METTL21C* | SSC11: 71021109 bp - SSC11: 71021201 bp  5’flanking | CpG1^†^ | 11:71021171 | 85.3824 | 86.8235 | 1.7×10^-3**^ | Up | -0.2329 | 5.6×10^−2^ |
|  |  | CpG2^†^ | 11:71021168 | 90.2941 | 91.2353 | 4.6×10^-2*^ |  | -0.5183 | 6.0×10^−6***^ |
|  |  | CpG3^†^ | 11:71021150 | 66.6471 | 70.0294 | 6.2×10^-5***^ |  | -0.3369 | 5.0×10^−3**^ |
|  |  | CpG4^†^ | 11:71021135 | 56.4706 | 60.2647 | 6.2×10^-5***^ |  | -0.4015 | 6.9×10^−4***^ |
| *PITX1* | SSC2: 137207285 bp - SSC2: 137207363 bp  5’flanking | CpG1 | 2:137207342 | 2.4118 | 2.2059 | 1.7×10^-1^ | Up | NA | NA |
|  |  | CpG2 | 2:137207329 | 2.8529 | 2.6471 | 5.1×10^-1^ |  | NA | NA |
|  |  | CpG3 | 2:137207326 | 3.7059 | 3.4706 | 5.1×10^-1^ |  | NA | NA |
|  |  | CpG4 | 2:137207313 | 4.8529 | 4.7059 | 8.3×10^-1^ |  | NA | NA |
|  |  | CpG5^†^ | 2:137207302 | 14.4412 | 16.1471 | 2.7×10^-2*^ |  | 0.1753 | 1.5×10^−1^ |
| *PVALB* | SSC5: 10955619 bp - SSC5: 10955739 bp  exon 1 | CpG1 | 5:10955644 | 23.3824 | 23.6176 | 8.9×10^-1^ | Down | NA | NA |
|  |  | CpG2 | 5:10955653 | 10.9706 | 11.1176 | 8.9×10^-1^ |  | NA | NA |
|  |  | CpG3 | 5:10955664 | 7.4118 | 7.3529 | 5.5×10^-1^ |  | NA | NA |
|  |  | CpG4 | 5:10955698 | 13.4412 | 13.8235 | 4.9×10^-1^ |  | NA | NA |
|  |  | CpG5 | 5:10955702 | 10.5000 | 10.6176 | 8.7×10^-1^ |  | NA | NA |
|  |  | CpG6 | 5:10955707 | 15.6765 | 15.5588 | 6.9×10^-1^ |  | NA | NA |
| *SIM1* | SSC1: 67294867 bp - SSC1: 67294976 bp  5’flanking | CpG1^†^ | 1:67294945 | 18.9706 | 14.2059 | 1.6×10^-2*^ | Up | 0.6393 | 4.4×10^−9***^ |
|  |  | CpG2^†^ | 1:67294938 | 16.5000 | 12.7647 | 1.1×10^-2*^ |  | 0.6536 | 1.5×10^−9***^ |
|  |  | CpG3^†^ | 1:67294934 | 9.0588 | 7.2353 | 4.9×10^-2*^ |  | 0.4505 | 1.2×10^−4***^ |
|  |  | CpG4 | 1:67294922 | 15.2941 | 11.3529 | 8.0×10^-2^ |  | NA | NA |
|  |  | CpG5 | 1:67294920 | 7.6176 | 6.2647 | 8.9×10^-2^ |  | NA | NA |
|  |  | CpG6 | 1:67294909 | 9.4412 | 7.9412 | 2.3×10^-1^ |  | NA | NA |
|  |  | CpG7^†^ | 1:67294900 | 7.4118 | 3.5000 | 1.6×10^-3**^ |  | 0.3844 | 1.2×10^−3**^ |
|  |  | CpG8 | 1:67294898 | 5.7059 | 7.0588 | 2.9×10^-1^ |  | NA | NA |
|  |  | CpG9 | 1:67294894 | 5.4412 | 6.5882 | 2.9×10^-1^ |  | NA | NA |
| *TBX1* | SSC14: 51289046 bp - SSC14: 51289196 bp 5’UTR | CpG1 | 14:51289069 | 86.0000 | 84.8529 | 8.1×10^-1^ | Down | NA | NA |
|  |  | CpG2 | 14:51289079 | 89.6765 | 89.8824 | 7.1×10^-1^ |  | NA | NA |
|  |  | CpG3^†^ | 14:51289109 | 74.5588 | 69.2941 | 4.9×10^-2*^ |  | -0.3933 | 9.1×10^−4***^ |
|  |  | CpG4 | 14:51289133 | 38.1177 | 38.8529 | 6.4×10^-1^ |  | NA | NA |
|  |  | CpG5 | 14:51289166 | 45.6471 | 46.9706 | 1.1×10^-1^ |  | NA | NA |

† Significant cytosines. rho: Spearman's rank correlation coefficient (ρ) is a nonparametric measure, that determines the strength and direction of the monotonic relationship between variable. Significant codes for p-value: * *p*<0.05, ***p*<0.01, ****p*<0.001

Table S4. The Spearman’s correlation coefficients between the RNA-seq count data and the qPCR results.

| **Gene** | **rho** | ***p*-value** |
| --- | --- | --- |
| *ALX1* | 0.8424 | 5.3×10^−9 ***^ |
| *EYA2* | 0.8103 | 5.8×10^−8 ***^ |
| *HOXA7* | 0.8702 | 4.2×10^−10 ***^ |
| *METTL21C* | 0.9043 | 2.3×10^−7 ***^ |
| *PITX1* | 0.9569 | 2.2×10^−16 ***^ |
| *PVALB* | 0.8750 | 4.8×10^−7 ***^ |
| *SIM1* | 0.9008 | 2.5×10^−7 ***^ |
| *TBX1* | 0.9301 | 1.1×10^−13 ***^ |

rho: Spearman's rank correlation coefficient (ρ) is a nonparametric measure, that determines the strength and direction of the monotonic relationship between variable. Significant codes for p value: * *p*<0.05, ***p*<0.01, ****p*<0.001.

Table S5. Impact of SNP variant on transcription factor binding affinities in the promoter region for *METTL21C*.

| **Transcription factor** | **Dissimilarity** | |
| --- | --- | --- |
|  | **Reference variant C** | **Alternative variant T** |
| STAT4 (T01577) | 4.41% | 2.94% |
| E2F-1 (T01542) | 10.52% | NA |
| TFII-I (T00824) | NA | 14.27% |

NA: Indicates that the particular transcription factor binding site is either present only in the reference variant or only in the alternative variant, meaning the factor is either lost or gained due to the variant

Table S6. The allele frequencies of the rs341151132 in *GALNT16*, rs690269483 in *PAOX*, rs334837422 in *RFTN1* and rs318378401 in *SLC9A1*.

| **Gene** | **Variant (rs ID)**  **and genomic localization** | **Allele** | **Frequency** | | ***P*-value** | **Odds ratio** |
| --- | --- | --- | --- | --- | --- | --- |
|  |  |  | **UH** | **C** |  |  |
| *GALNT16* | rs341151132  Ref:C > Alt:T  SSC7: 93017882 bp | C | 0.81 | 0.69 | 0.1159 | \|  \| 0.5290 \| \| --- \| --- \| |
|  |  | T | 0.19 | 0.31 |  |  |
| *RFTN1* | rs334837422  Ref:C > Alt:T  SSC13: 3524357 bp | C | 0.74 | 0.81 | 0.3085 | 1.5231 |
|  |  | T | 0.26 | 0.19 |  |  |
| *PAOX* | rs690269483  Ref:A > Alt:G  SSC14: 141364546 bp | A | 0.65 | 0.75 | 0.1925 | 1.6364 |
|  |  | G | 0.35 | 0.25 |  |  |
| *SLC9A1* | rs318378401  Ref:A > Alt:G  SSC6: 84372805 bp | A | 0.24 | 0.32 | 0.2531 | 1.5543 |
|  |  | G | 0.76 | 0.68 |  |  |

Ref: Reference; Alt: Alternative


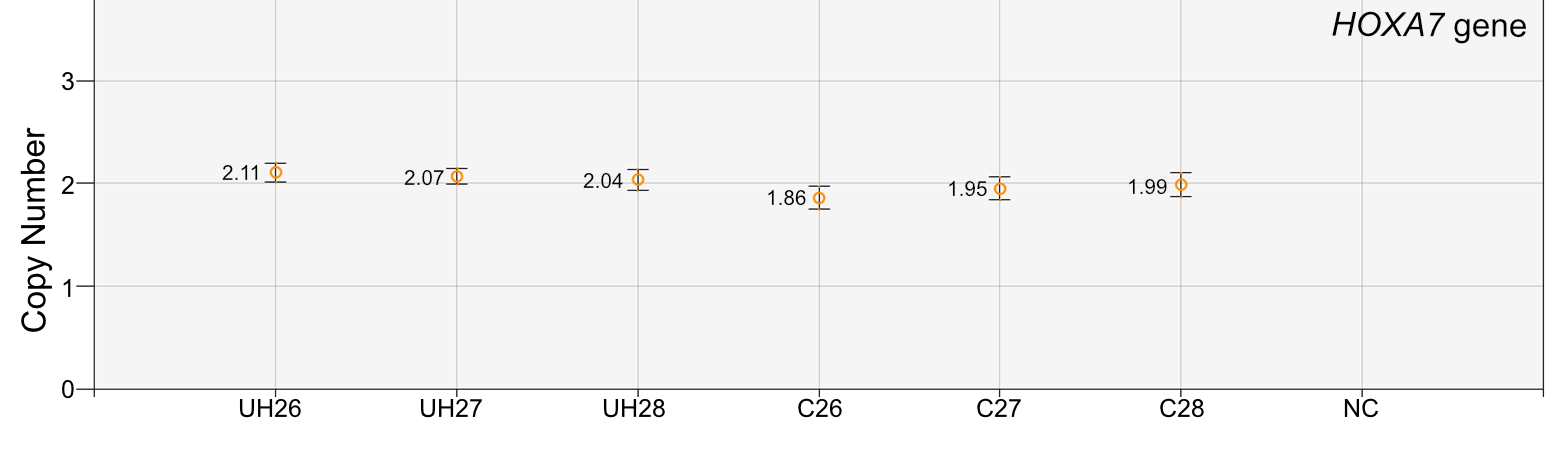


Figure S1. Identification of 2 copies of *HOXA7* gene in three UH cases and three control animals by ddPCR. NC - negative control (no DNA). Error bars represent the 95% confidence interval.


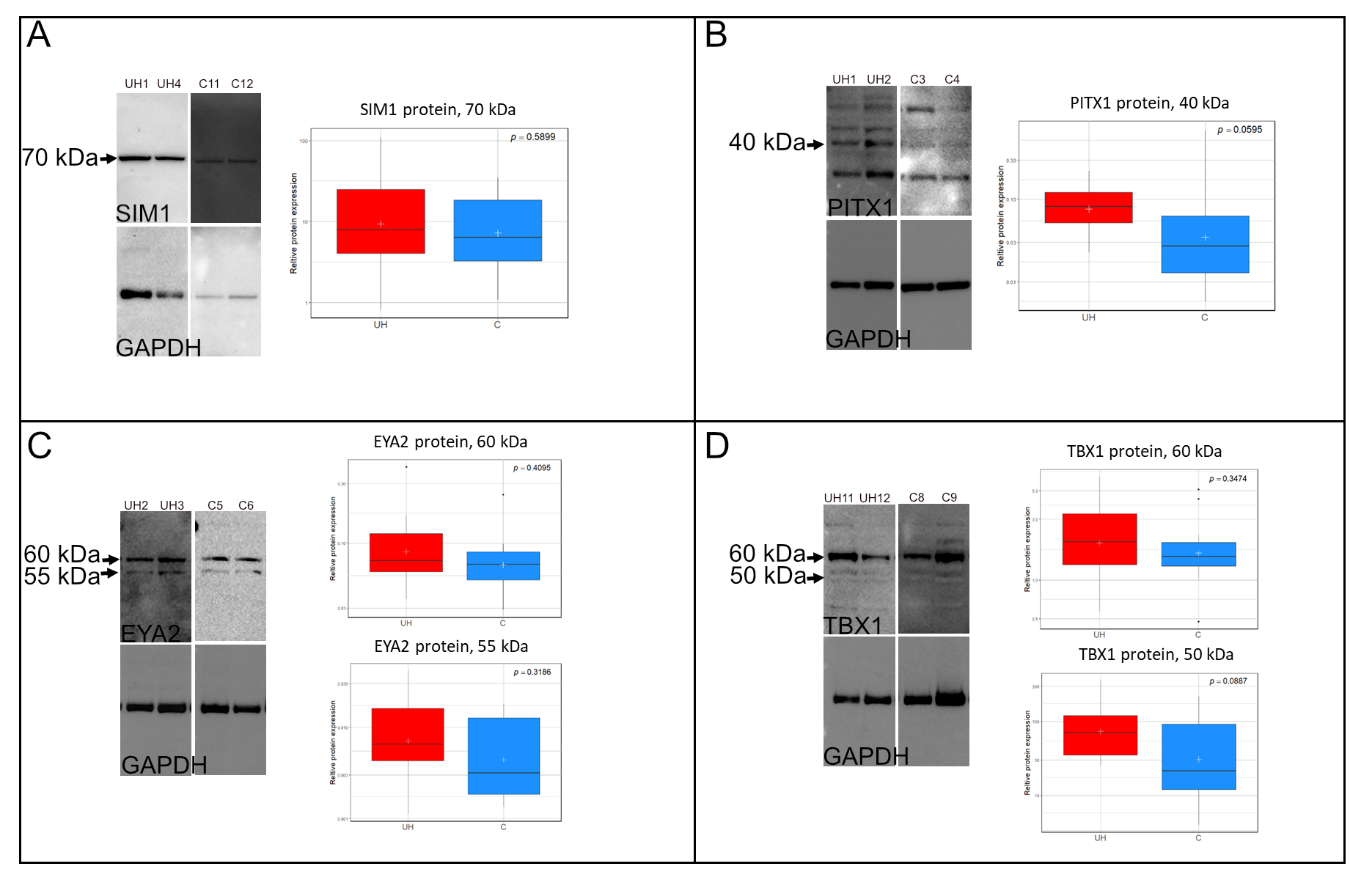


Figure S2. Protein level in UH and control animals - Western blot results for studied proteins in relation to GAPDH (reference) and relative expression in studied groups. A – results for SIM1, B - results for PITX1, C - results for EYA2 and D - results for TBX1. The vertical black lines crossing the boxes show medians and the white crosses represent mean values. The lines below and above the rectangles indicate the maximum and minimum values, while the black dots positioned beneath and above the boxes represent outliers. Original blots are presented in Figure S3.


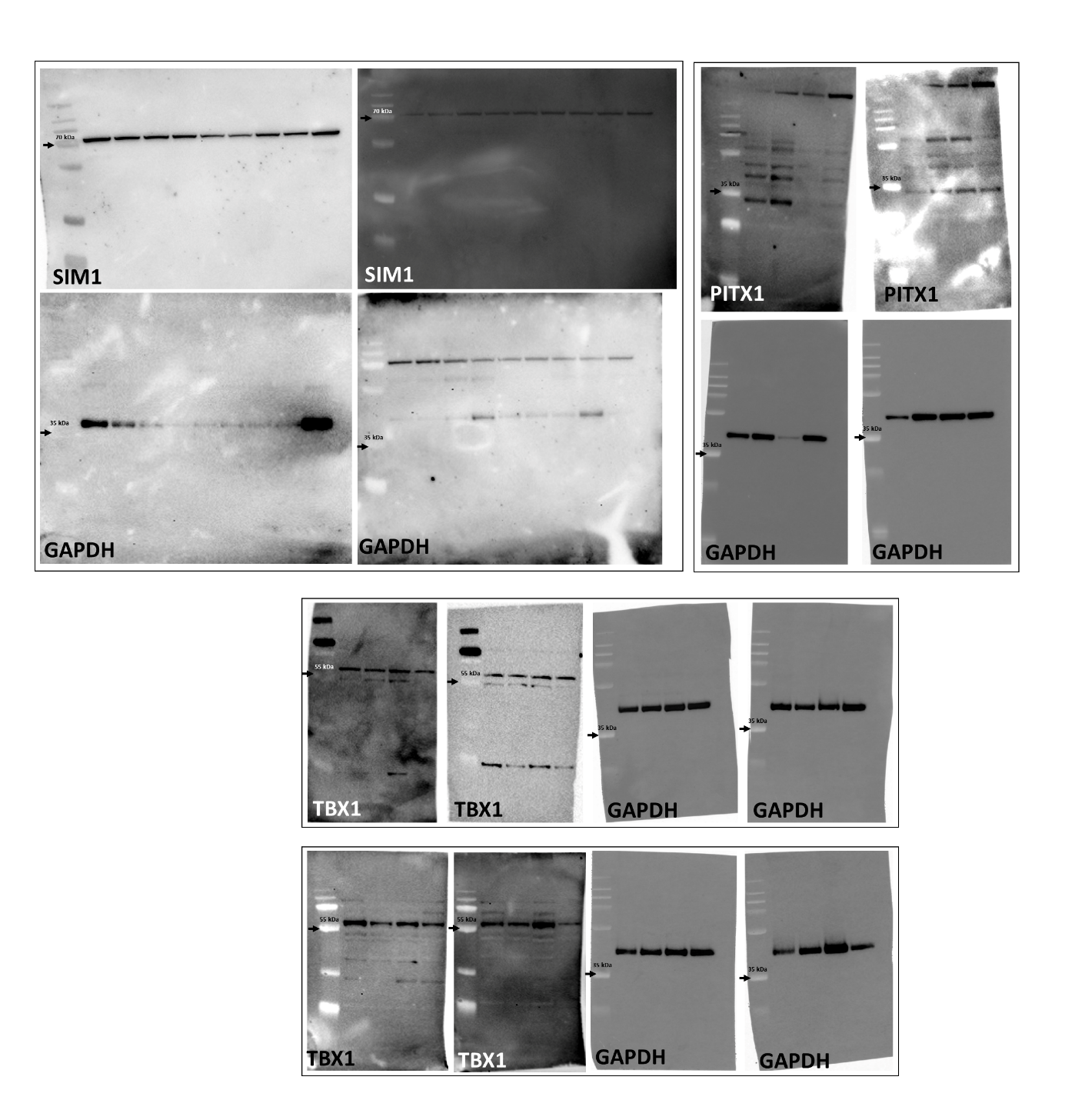


Figure S3. Unprocessed western blots gels. Arrows indicate the particular ladder bands.

Additional file 2 (xlsx): List of genes generated by differential expression analysis of RNA-seq data for the UH vs. C comparison

Additional file 3 (xlsx): List of significant GO terms for UH vs C comparison (for up- and downregulated genes)

Additional file 4 (xlsx): List of SNPs detected in RNA-seq analysis
